# DNA barcoding reveals a mysterious high species diversity of conifer-feeding aphids in the mountains of southwest China

**DOI:** 10.1038/srep20123

**Published:** 2016-02-03

**Authors:** Rui Chen, Li-Yun Jiang, Jing Chen, Ge-Xia Qiao

**Affiliations:** 1 Key Laboratory of Zoological Systematics and Evolution, Institute of Zoology, Chinese Academy of Sciences, No. 1 Beichen West Road, Chaoyang District, Beijing 100101, P.R. China; 2 College of Life Sciences, University of Chinese Academy of Sciences, No. 19, Yuquan Road, Shijingshan District, Beijing 100049, P.R.China

## Abstract

The mountains of southwest China are one of the hot spots of biodiversity in the world. However, the high-altitude fauna that inhabit these mountains remain a mystery. In this study, the species diversity of the aphids of the genus *Cinara* from the high-altitude coniferous forests was first assessed, and then the processes and the mechanisms of speciation were discussed. Three hundreds and four aphid samples that contained 3040 individuals were collected during fourteen field surveys. The molecular clusters derived from the DNA barcodes were used to explore the species diversity. Notably, the aphid alpha-diversity was high, with as many as 94 candidate species, and furthermore, 86.2% of the species collected had not been previously recorded. The centers of aphid species richness corresponded to the distributional pattern of the diversity of the host conifer plant species. The divergence time revealed that following the uplift of the Qinghai-Tibetan Plateau during the Pleistocene, the changes in the climate, ecology and host habitats were likely the most important factors that drove the rapid process of evolutionary radiation in the aphids. Our findings revealed the high species diversity of the aphids with DNA barcoding.

The mountains of southwest China occupy the area between the easternmost edge of the Qinghai-Tibetan Plateau (QTP) and the Central Chinese Plain and are recognized as one of the 34 hot spots of biodiversity in the world (http://www.biodiversityhotspots.org/xp/Hotspots). Based on the unique fauna, this region has long been considered an independent realm in zoogeography^1^,^2^. Therefore, the determination of the species diversity and the distribution patterns of diversity are important to gain a better understanding of the mechanisms that formed and help to maintain the biodiversity of this hot spot^3^. However, the biodiversity surveys in the high-altitude zones of these mountains face major challenges, because many of the regions are unknown or have rarely or never been visited. In the mountains of southwest China, the coniferous forest is the primary vegetation type in the high-altitude zones. However, studies have rarely focused on the insect species diversity in these high-altitude coniferous forests.

The identification of species based on morphology is frequently used to explore biodiversity. However, such morphology-based taxonomy is often difficult, particularly for a community with largely undescribed biodiversity^4^. DNA barcoding has become an important tool in the development of a global inventory of biodiversity^5^,^6^. The automated DNA-based approaches for OTU (operational taxonomic units) designation have demonstrated their usefulness in probing biodiversity^7^ and have important advantages^8^. To estimate biodiversity, DNA barcoding has been successfully used in many insect groups, including ants^9^, flies^10^ and wasps^11^.

Aphids are phloem-feeding insects in the order Hemiptera, with more than 5000 species^12^. The range of the host plant restricts the distribution of most aphids. As the primary genus of aphids that feeds on conifers, *Cinara* Curtis is the second largest genus of aphids with 243 described species^12^; the aphids in this genus feed exclusively on conifers primarily in the temperate and subtropical regions of the Northern Hemisphere. The host plants for this genus of aphid are found in several genera, including *Abies, Picea* and *Larix*, which have broad geographic distributions and patterns of species diversity from 2500 to 4500 m in elevation within this region^13^. The species of *Cinara* have disjunctive distributions and different allopatric populations because of the specific geographic ranges of the host plants. The dispersal ability of species of *Cinara* is limited because of the high weight to wing length ratio^14^, and some species are even recorded without winged morphs^15^; the absence of dispersal ability might accelerate the genetic diversification of species in an area with such complex topography^16^. However, previous investigations on aphids in the high-altitude zones of the mountains of southwest China are rare.

In this paper, we first assessed the aphid diversity and focused on the species in the coniferous forests of the high-altitude zones with field surveys in the last ten years, particularly during three targeted expeditions in 2012–2013. We then used the molecular clusters that were derived from the DNA barcodes to delineate putative species and to explore the diversity of aphids in the mountains of southwest China. The aims of the research were as follows: 1) to identify the genetic diversity of the aphids and to determine the ratio of described to undescribed species, 2) to describe the distribution patterns of the species and to assess the roles of two factors (elevation and host plants) on the patterns of distribution, and 3) to estimate evolution of the host associations within the aphid genus and to discuss the processes and mechanisms of speciation for the aphid genus in this region.

## Results

### Taxonomic assignments and species delineations

A 658-bp PCR product for the COI gene was obtained from 304 samples, among which 373 sites were conserved, 285 were variable and 262 were parsimony-informative. These sequences were heavily biased toward A and T nucleotides, averaging 39.1% for T, 14.9% for C, 35.6% for A and 10.4% for G nucleotides.

Both the clustering method based on the NJ tree and the genetic distances and the ABGD method identified 94 candidate species from the 304 barcodes (Table S1). GMYC model provided evidence of 94(86–101) independent entities. Therefore, we defined 94 candidate species in this research, and among these candidate species, 48 species (51.1%) were discovered at only one sample site, whereas 8 species (8.5%) were distributed in more than ten sample sites (Table S1).

Based on morphological taxonomy, only 13 candidate species from 114 samples were successfully identified to the 13 *Cinara* known species. Among these species, *Cinara cuneomaculata* (del Guercio) and *Cinara piniarmandicola* Zhang, Zhang & Zhong were first discovered in this region, whereas seven species that were previously recorded in the region were not found (Table S2). When using our DNA bar-code database, 114 DNA barcodes were matched to the 13 *Cinara* species, which was consistent with the morphological identifications. However, based on these two methods, the remaining 81 candidate species did not fit the description of any known *Cinara* species. These species had morphological differences with the known *Cinara* species, although some of the species shared some near-morphological characters with certain known species. Notably, most of the identified species (9/13) were widely distributed in the mountains of southwest China and were found at relatively low elevations (1500–3000 m), whereas many of the undescribed species (33/81) were found at relatively high elevations (above 3500 m).

### Mapping species diversity

**Figure 1:**
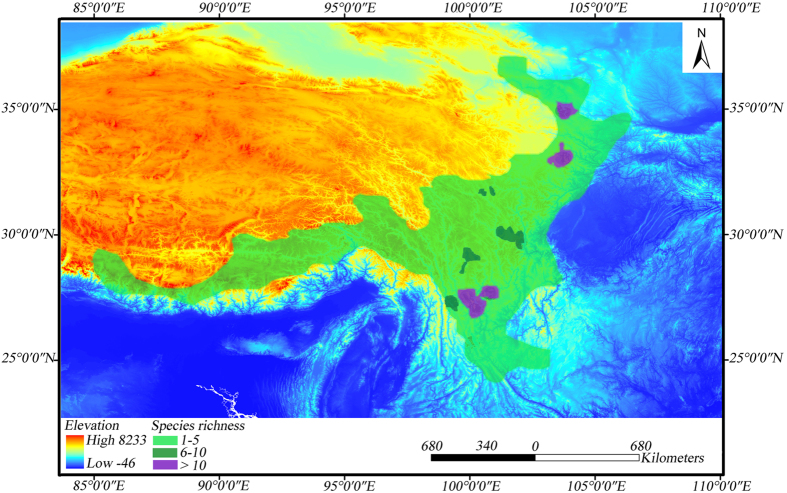
Map of species richness of *Cinara* in the mountains of southwest China. This original map was created using ArcGIS 9.3 software.

Based on the 94 *Cinara* species/candidate species that were identified, the species diversity of *Cinara* in the mountains of southwest China was concentrated in three areas (Fig. 1): (1) the mountainous areas associated with the Sichuan-Gansu border, the western Qinling and southern Gansu Province, (2) the northern Hengduan Mountains region, and (3) northwestern Yunnan Province. These three areas were the centers of distributions in the mountains of southwest China and had the highest species diversity.

### Patterns of *Cinara* species richness along the elevational gradients

**Figure 2:**
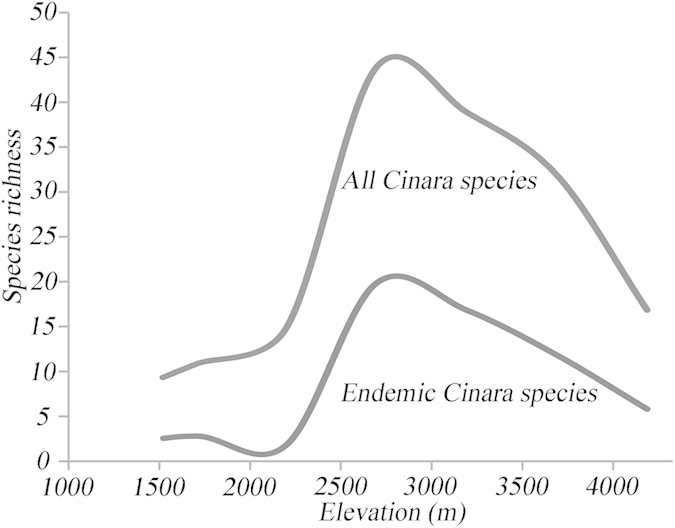
Distribution pattern by elevation of the species richness of *Cinara* in the mountains of southwest China.

The patterns of species richness with elevation in the mountains of southwest China for all *Cinara* species/candidate species are shown in Fig. 2. The total species richness was highest at the medium elevations (2500–3000 m), and the endemic species richness was also the highest at the 2500–3000 m elevation.

### Patterns of *Cinara* species richness in relation to different host plants

**Figure 3:**
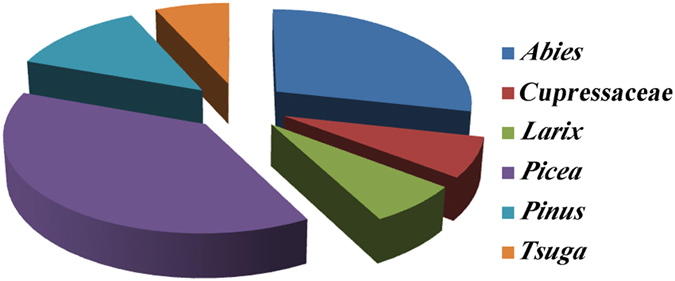
Species richness for the different host plant taxa of *Cinara* in the mountains of southwest China.

The host plants for the species of *Cinara* included *Abies*, Cupressaceae, *Larix, Picea, Pinus* and *Tsuga* (Fig. 3). More than one-third of the species of *Cinara* lived on *Picea*, and *Abies* was also inhabited by many of the *Cinara* species. The *Cinara* species richness on *Larix, Tsuga* and Cupressaceae was relatively low (6, 6 and 9, respectively).

### NJ Tree Structure and COI divergence assessment

**Figure 4:**
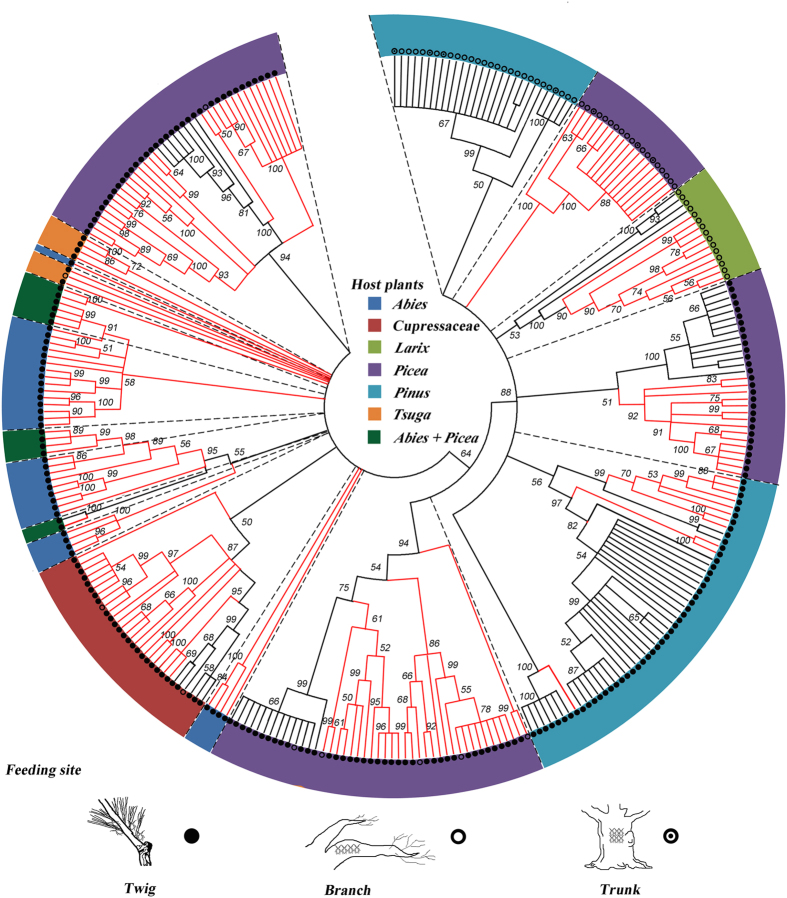
Neighbor-joining tree (K2P distance) of the *Cinara* species in the mountains of southwest China. Only the bootstrap values greater than 50 are shown. The colors represent different host plants, and the red regions represent new species/candidate species lineages.

The results of the overall NJ analysis by COI region of the distances among the 304 samples are shown in Fig. 4. The trees represented only the distance matrix and should not be interpreted as phylogenetic hypotheses. Of the *Cinara* species, almost all were clustered according to a specific host plant. Of the aphids on the different genera of host plants, almost none were found together except for those on *Abies* and *Picea*.

For the genetic divergence along the different altitudinal gradients (Table 1), the elevation zone at 3500–4000 m had the highest interspecific divergence (0.1082 ± 0.0243), which was followed by the elevation zones that exceeded 4000 m (0.1062 ± 0.0212) and the 2500–3000 m elevation zone (0.1029 ± 0.0217). Additionally, all elevation zones had low levels of intraspecific variation (0.0005 ± 0.0012 to 0.0025 ± 0.0027).

For the genetic divergence affected by different ecological variables (Table 2), the variable sets concerning allopatric/different feeding site/hosts in the different genera displayed the highest interspecific divergence (0.1352 ± 0.0203). The sets concerning allopatric/different feeding site/hosts in one genus and the sets concerning sympatric/different feeding site/hosts in different genera also showed high interspecific divergence. By contrast, the sets concerning allopatric/same feeding site/hosts in one genus and the sets concerning allopatric/same feeding site/hosts in different genera yielded higher intraspecific divergences than the others.

### Divergence times

**Figure 5:**
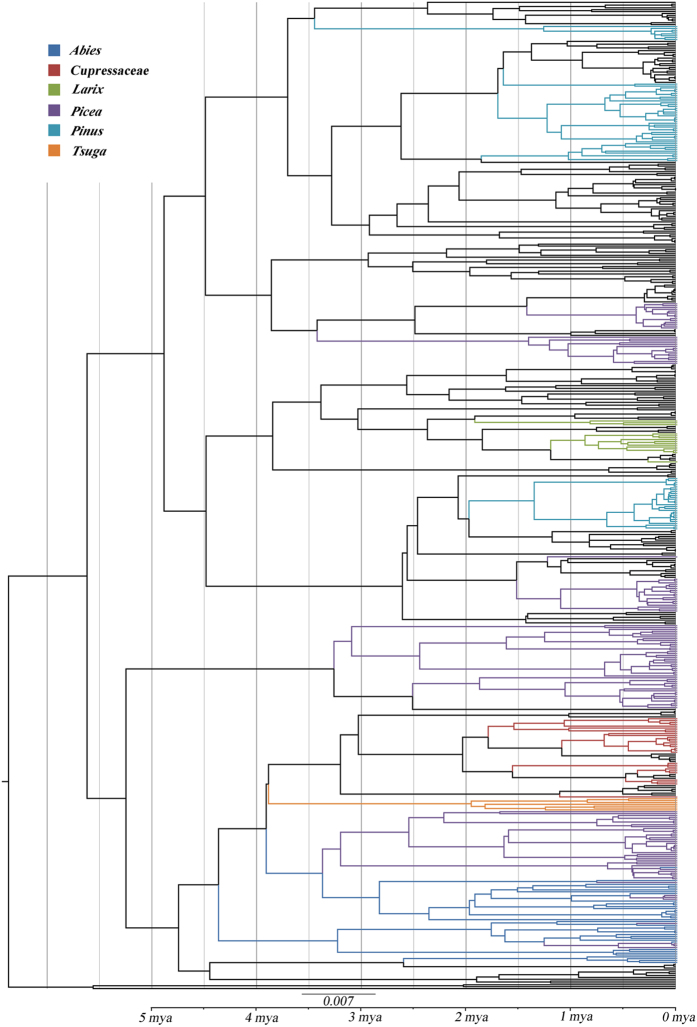
A time-calibrated phylogenetic tree resulting from the BEAST analysis. The color clusters represent *Cinara* samples from the mountains of southwest China, and the black clusters represent *Cinara* samples from Europe and North America. The colors represent different host plants.

The ultrametric tree with the divergence times estimated from the BEAST analysis is shown in Fig. 5. All species-level divergences in *Cinara* occurred less than 3.5 Ma. The *Cinara* species in this region fed on *Abies* and *Picea* early before 3 Ma and then diversified onto different hosts ca. 2 Ma. These results suggested that there were multiple acquisitions of hosts by the *Cinara* in the mountains of southwest China, including at least five from *Pinus*, 11 from *Picea*, three from *Larix*, four from *Abies*, one from *Tsuga* and six from Cupressaceae.

### Diversification rates

The LASER results rejected the null hypothesis of temporally homogeneous diversification rates and suggested variable diversification rates within *Cinara*. The diversification rate-constancy statistic ΔAIC_RC_ was 15.89. The yule2rate diversification model was selected as the best fitting model. The Relative Cladogenesis (RC) analyses detected significant shifts of diversification rates within *Cinara* at ca. 4.35 Ma.

## Discussion

### High diversity of conifer-feeding aphids in the mountains of southwest China

Our study revealed high genetic diversity of the conifer-feeding aphids in the mountains of southwest China based on species delineation (94 candidate species). The species-level identifications were based on morphological diagnostic features and DNA barcoding, which allowed us to identify 37.5% of the samples (114 of 304 aphids) as species among the 13 known species. However, 86.2% of the candidate species did not fit the description of any known species. Therefore, our results established unequivocally that most of the conifer-feeding aphid species in this region were unknown and undescribed, with a large number of the species documented in this study for the first time.

Significantly, many of the sample locations, which were rarely or never visited, for the conifer-feeding aphid species were in large and unknown regions at elevations above 3000 m in our study area. For example, 34 specimens were collected from the elevation zones over 4000 m, and the highest sample elevation was 4782 m. Previously, no conifer-feeding aphid species were found above 4000 m in this region^2^. The high-elevation “sky island” populations might have remained isolated with little opportunity for gene flow. Additionally, according to previous publications and museum records, 18 *Cinara* species were distributed in this region, and only 6 species were described for *Picea* or *Abies*. However, based on our data, more than half of the *Cinara* species were found on *Picea* and *Abies*, which were patchily distributed in the high elevation zones (2500–4000 m) and displayed high diversity^13^. Therefore, the species richness was unusually high in this region for the *Cinara* species. This study, based on the novel barcodes, provided a new evidence to evaluate the species richness in the high elevation zones.

### The three centers of distribution were predicted

With the uplift of the QTP, the topography and the climate of the mountains of southwest China changed significantly^17^. An uneven distribution of vegetation and an extremely complex topography codetermined the spatial patterns of aphid diversity in this region^2^. Three distribution centers for the *Cinara* species were found in this region: (1) the associated mountainous areas on the Sichuan-Gansu border, in the western Qinling and in southern Gansu Province, (2) northwestern Yunnan Province, and (3) the northern Hengduan Mountains region.

As one distribution center of the *Cinara* species, the associated mountainous areas on the Sichuan-Gansu border, in the western Qinling and in southern Gansu Province are transitional areas from the main body of the plateau to the lower hilly terrains. These areas are the regions that divide the Palearctic and Oriental Realms in central China^18^, which also have a high diversity of plants. A high abundance of *Abies* was found in this region^13^, which provided opportunities for the *Cinara* species to diversify. Similarly, another center, in northwestern Yunnan Province, is also located in the transitional zone between the Palearctic and the Oriental realms^19^,^20^ and has the highest diversity of plants in the Hengduan Mountains. This region was proposed as a refuge during the Pleistocene glaciations^18^,^21^. The *Cinara* that inhabited this region had high levels of species richness because presumably the high plant diversity and the Pleistocene conditions promoted the differentiation and the speciation of the aphids. The third center of *Cinara* species is in the northern Hengduan Mountains region, which has many mountain ridges and deep gorges. Compared with the rest of the Hengduan Mountains, the northern Hengduan Mountains ranged from 2500 to 4000 m in elevation and formed the center for *Abies* and *Picea* pollen abundance^13^.

From these patterns, we concluded that the centers of distribution of the conifer-feeding aphid species strongly corresponded with the pattern of conifer species richness, particularly for *Abies* and *Picea*.

### Elevational and host associated patterns of conifer-feeding aphid species richness

The elevation patterns for the total and the endemic species richness of the aphids displayed unimodal patterns, which was similar to previous reports for plants^22^,^23^. The *Cinara* patterns of species richness, for all species and endemics, were highest at the middle elevations (2500–3000 m). Some previous studies highlighted that the peak for endemic species richness was at a higher elevation than the peak for total species richness^24^,^25^, whereas other studies found that the total and the endemic species richness peaked at the same elevation^23^, which was consistent with our results. Our study area, the mountains of southwest China, provided a refuge for many temperate plant taxa during the Pleistocene. Associated with the ever-changing habitat of the rising plateau during the postglacial periods, the plants in this area experienced rapid evolution^26^. During the alternating processes of glaciation and deglaciation, the middle elevations were more likely to be the center for repeated plant migrations between the high and the low elevations^23^. Additionally, the middle elevations provided a moist, shady environment for coniferous plants and were not subject to the sharp fluctuations in weather that occurred throughout the year at the higher elevations or to the drying effects of the rain shadows at lower elevations^23^. Therefore, the suitable conditions for high plant diversity in the coniferous forests of the middle elevations provided more suitable ecological niches for the aphids. Most of the *Cinara* species evolved in this region and some were limited to only a single mountain (according to our field investigations, unpublished data). The rapid speciation in *Cinara* resulted in more than one-third of the species restricted to this region, and therefore, the expectation was reasonable that elevation patterns for the total and the endemic species richness were strongly correlated.

The species richness of the genus *Cinara* was diversified in relation to the different host taxa. The number of sample sites on different hosts was not associated with species richness; for example, 78 sites were on *Pinus* in the mountains of southwest China, but only 13 species/candidate species were discovered on this host plant genus. As a comparison, eight samples were on *Tsuga*, which had seven species/candidate species. In the mountains of southwest China, *Pinus* is primarily distributed in the relatively low elevation areas (1500–2500 m), and therefore, this genus might have had little effect on the complex topography of the *Cinara* species that inhabited this region. By contrast, *Tsuga* primarily occurs in the 3500–4000 m elevation zones and is distributed sporadically. Thus, *Tsuga* would provide the geographic isolation and the host isolation that prevented gene flow among the different aphid populations, which would lead to genetic divergence. Additionally, the *Cinara* on *Picea* and *Abies* displayed high species richness. The species richness of *Picea* and *Abies* is high in the high elevation zones (2500–4000 m) of the mountains of southwest China^13^. Thus, the complex topography, the suitable microenvironmental conditions, and the high diversity of *Picea* and *Abies* provided opportunities for the *Cinara* species to diversify and to speciate. Furthermore, the *Cinara* species on the Cupressaceae and the *Larix* formed genetically distinct lineages that were related to geographical location. The range area is an important explanatory variable for the patterns of species richness^27^. The distributions of Cupressaceae and *Larix* in the mountains of southwest China ranged from low elevations (1500 m) to high elevations (4500 m) and exhibited a wide disparity in height. Thus, the local vicariance and complex topography along the elevation gradient were major factors that affected the species richness of aphids.

### Rapid evolutionary radiation of the *Cinara* species

Our analysis suggested variable diversification rates within *Cinara* and shifts of diversification rates at ca. 4.35 Ma. The first acquired hosts of *Cinara* were *Picea* and *Abies*, and the separation of these lineages from the other lineages was assumed to occur ca. 3‒4.5 Ma, following the first and the second phases of the “Tibet movement.” The “Tibet movement” occurred between 3.6 and 1.7 Ma and included three phases that commenced at 3.6, 2.5, and 1.7 Ma^28^. During this period, the regional environment was relatively warm and humid, and there was a subtropical mixed forest of evergreen broadleaf and broadleaf deciduous woodlands^29^; there were no coniferous forests and only a few fir and spruce trees^13^. Thus, the early *Cinara* species fed only on *Picea* and *Abies* and maintained a low diversity. The early speciation within *Cinara* in this region was estimated at ca. 2–3.5 Ma, and a more large-scale rapid speciation was estimated to occur at less than 2 Ma. This speciation event occurred approximately at the time of the third phase of the “Tibet movement” and the “Kunhuang movement,” when the plateau was uplifted to an average height of 3000 m, with mountains up to and over 4000 m^30^. The uplift of the Tibetan Plateau and the surrounding areas caused a climatic and ecological shift; the evergreen broadleaf and broadleaf deciduous forests were replaced with coniferous forests and grasslands, the climate gradually became drier, colder, and windier, and the development of the glaciers began^29^. Since the uplift, the plateau has undergone several glaciations^31^. The dramatic climatic and environmental changes caused by the uplift of the Tibetan Plateau in the Pliocene-Early Quaternary resulted in new habitats for many gymnosperms, which facilitated the evolution of the *Cinara* species. Therefore, the Pliocene-Early Quaternary might be the period in which the adaptive radiation of *Cinara* reached a maximum in this region. The pronounced mountain formation at that time led to an isolation of the populations and the formation of many “mountain-island” species.

### Speciation of *Cinara* in the mountains of southwest China

The processes and mechanisms of speciation were influenced by multiple driving forces^32^. Most speciation results from isolation combined with divergent selection, such as selection on ecological traits^32^. In the classic allopatric model of Mayr (1947), different ecological conditions drove the speciation in geographically isolated populations^33^. Our analyses on the conifer-feeding aphid genus, *Cinara*, revealed that the high diversity of these aphids was strongly associated with the ecological conditions in the mountains of southwest China. The host plant was an important ecological factor that affected the species richness of *Cinara*. The different *Cinara* species had different feeding preferences, and most species fed on a single or a few closely related host species, whereas others fed on several species within a genus and sometimes even on hosts in different genera of conifers (i.e., some species inhabited either *Picea* or *Abies*). Based on our analysis of the genetic divergence (Table 2), the intra- and interspecific divergence of aphids on hosts of different genera were all greater than those of aphids on a host in one genus, which indicated that closely related species of *Cinara* tended to feed on closely related host species.

Additionally, the *Cinara* species had specific feeding sites on the host plants. Some species fed only on young twigs, whereas others fed exclusively on old branches or large trunks^34^. For the *Cinara* species in our study, the aphids that used similar feeding sites were more closely related than the aphids that used different feeding sites (Fig. 4, Table 2). Additionally, the species that used similar feeding sites on hosts from different genera were less divergent than the species from different feeding sites on one host (Table 2). These results provided evidence that the speciation in some *Cinara* species in the mountains of southwest China occurred through a shift to new hosts. Similarly, Favret & Voegtlin (2004) speculated that the *Cinara* species apparently adapted more easily with a switch to a similar microhabitat on a different host than to feeding on a different part of the same tree species^35^.

Furthermore, the complex topography in the mountains of southwest China had a strong effect on the acceleration of the diversification of the *Cinara* species. Our data revealed that the intraspecific divergences from allopatric populations were significantly greater than those from sympatric populations (Table 2). Additionally, the species that inhabited the high elevation zones (>2500 m) had higher interspecific divergence than those from the low elevation zones (<2500 m) (Table 1). These results demonstrated that geographic isolation promoted speciation events in *Cinara* and that geographical barriers such as high mountains appear to be ‘isolated islands’ that shaped some of the diversity at regional scales.

Consequently, the high diversity of *Cinara* in the mountains of southwest China was caused by a combination of factors, which included host diversity, a complex topography and historical climatic oscillations. Geographic isolation played a driving force that caused the populations to differentiate, whereas a shift in hosts was a major factor that affected species-level divergence. Ultimately, the host diversity and the niche diversification caused by the uplift of the Tibetan Plateau in the Pliocene-Early Quaternary caused the high species richness of the genus *Cinara* in this region.

In this study, the patterns of species richness of the genus *Cinara* in the mountains of southwest China helped us to understand the abundance of *Cinara* species. The genus *Cinara*, a conifer-feeding member in the Lachninae, is the key taxon to determine whether the conifer-feeding was ancestral to the extant lachnids, which had the dispute before^36^. A recent study has suggested that the host of the Lachninae ancestor was an angiosperm and that the diversification of *Cinara* is concomitant with the adoption of conifer hosts^39^. Based on our results, a large number of the *Cinara* species in the mountains of southwest China appeared after the uplift of the Tibetan Plateau and then experienced rapid speciation, which gave a new evidence for the evolution of host affiliation in the lachnids specifically.

## Methods

### Study area

The mountains of the southwest China hot spot cover over 262,400 km^2^, and the elevations range from less than 2000 m in some valley floors to 7558 m at the summit of Gongga Mountain. The geographical distributions of the aphids corresponded with the distributions of the conifers. The conifers were absent because of the environmental conditions in some of the mountainous areas. Thus, the area defined for this study was a relatively crude geographical range that corresponded to the distributional ranges of the conifers.

### Known species category of *Cinara* in the mountains of southwest China

Only 18 species of *Cinara* were described for the mountains of southwest China (Table S2), according to the statistics of Huang *et al*. 2006 2, the published literature^40^,^41^,^42^,^43^,^44^,^45^ and the information from specimens examined from the following museums: the National Zoological Museum of China, the Institute of Zoology, the Chinese Academy of Sciences (Beijing), the Natural History Museum (London), the French National Museum of Natural History (Paris), and the USDA Systematic Entomology Laboratory (Maryland). Among these described species, ten species fed on *Pinus*, six species fed on *Picea*/*Abies*, one species fed on *Larix* and one species fed on Cupressaceae.

### Field sampling and data collection

The fieldwork in the mountains of southwest China occurred during four targeted expeditions (April 20, 2006-May 16, 2006, for 27 days; May 21, 2012-June 2, 2012, for 13 days; June 20, 2012-August 9, 2012, for 51 days; and August 5–24, 2013, for 20 days) and on ten general expeditions (July 2002; August 2003; October 2003; August 2004; April-May 2005; September 2005; June-July 2009; November 2009; August 2010; and October 2010) at different times of different years to ensure that the samples covered the entire geographical range of distribution. Because of the unique geographical landforms and the distribution ranges of the host plants, all sample sites were above 1500 m. To avoid the effects of artificial forestation, we tended to select samples from the original forest tree species instead of the planted street and garden trees. For each sample site, we attempted to sample as many species as possible from one or more different host species. The host trees were identified to the genus of conifer and to the species, when possible, using the local flora. Because of the special reproductive mode of aphids (parthenogenesis), a species on a specific host within a certain geographic range might belong to the same clone. Thus, a species on the same host plant at the same sample site within a 10-kilometer radius was sampled only one time, which was regarded as one sample, unless some individuals displayed some differences in biology. In total, we collected 3040 individual aphids from 304 samples (10 individuals per sample), and at least 5 individuals of each sample were sequenced before preparation to ensure that the samples did not contain multiple species and all individuals in one sample belong to the same clone. One to two representative individuals from each clone were sampled for the molecular analyses.

### Species delineation

The total DNA was extracted from a single aphid that was preserved in 95% or 100% ethanol, and the amplicon size of the COI was approximately 660 bp. The multiple alignments were generated using CLUSTALX^46^, which were subsequently pruned to lengths of 658 bp (COI). The sequences were deposited in the GenBank, and the accession numbers are provided in Table S1.

The species delineation was based on the analysis of the DNA barcodes with automated procedures. Many methods to use DNA data to delimit species were recently proposed^6^,^47^, and in this study, we chose three methods for the species delineation.

First, we applied a simple clustering method^48^, with some improvements. We only considered a ‘candidate species’ when (i) the ‘candidate species’ was in the COI-based NJ tree reconstructions and (ii) the clustering was strongly supported in the COI-based NJ tree (bootstrap support>70). The clades that were identified were well differentiated from one another to prevent minor tip clades from being recognized as distinct species. We applied a threshold difference of at least two percent in the COI sequences between individual specimens of a given clade and those of the nearest branch (a 2% difference in the COI sequence is the smallest difference that is generally found between unambiguously identified aphid species^49^).

The second method we used was the Automatic Barcode Gap Discovery (ABGD)^50^. This method was used to objectively delimit the operational taxonomic units considered in this study to be proxies for species, although only an integrative approach (one that combined thorough morphological comparisons with additional markers) could assess the distinctions between species. The results of the ABGD were dependent on the maximum p-threshold that was selected a priori in the group that was studied.

The third method we used was the generalized mixed Yule coalescent (GMYC) model^51^. The method uses a maximum likelihood approach to optimize the threshold identifying the shift in the branching patterns of the gene tree from interspecific branches (Yule model) to intraspecific branches (coalescent). It identifies clusters of sequences corresponding to independently evolving entities. The maximum likelihood approach also provides a 95% confidence interval around the maximum likelihood solution. We used the conservative approach of identifying the minimum number of entities within the 95% confidence interval. The GMYC model was performed with the R package *splits* 1.0–11 (https://r-forge.r-project.org/projects/splits/).

Finally, we defined a ‘candidate species’ which was supported by most methods of species delineation. To easily clarify the scientific definition of a species, the ‘candidate species’ defined in this study were considered ‘species’ in our analysis. However, the ‘candidate species’ were not necessarily true species because the genetic diversity was displayed only at the molecular level. The description of a new species requires many hours of morphological work and should not rely only on DNA data.

Based on the results from the species delineations, we used diagnostic morphological features to identify the species. Three to five individual aphids per collection location were mounted on microscope slides for morphological examination. The voucher specimens for each sample were identified by Qiao, who based the identifications on diagnostic morphological features using standard literature-based keys^15^ and by the comparison with previously identified specimens in the National Zoological Museum of China, Beijing. The samples and the voucher specimens were deposited in this same museum. The complete list of the taxa with the collection data, including the host plants, Feeding sites and the dates, are provided in Supporting Information, Table S1.

Because of the taxonomic impediments and the difficulties of delimiting the morpho-species from a large set of samples in the field, we also identified species with a comparison of the DNA barcodes used in this study with our aphid DNA bar-coding database, which stored large numbers of DNA barcodes from our previous studies^52^. We compared the DNA barcodes to determine whether the samples matched any known aphid species.

### Mapping the distribution pattern

Based on the database from our field collections, we used ArcGIS 9.3 (ESRI co., USA) to map the distribution of the *Cinara* species in the mountains of southwest China (Figure S1). Additionally, to determine the centers of diversity, a density map of the *Cinara* species was created with an overlay of the distribution of the candidate species that we defined and the GIS layer of the mountains of southwest China (Fig. 1). To obtain more objective patterns of distribution, the distribution of each species/candidate species was limited to a certain altitudinal range according to the locations of the collection sites and the altitudinal distributions of the host plants (Table S3). The species richness on the map was then estimated based on statistical calculation.

### Data Analyses

We divided the range of elevations into 500-m bands between 1500 and 5000 m, and we calculated the total number of species/candidate species and the number endemic species (defined as the species/candidate species collected only from one site or from one relatively small area) in each band. Additionally, the total numbers of species/candidate species with the different host plant taxa were calculated. Moreover, the average interspecific and intraspecific divergences along different altitudinal gradients with the effects of different environmental variables were calculated using the Kimura 2-parameter model (K2P). The environmental variables included three factors: allopatric or sympatric populations, the same or different feeding sites, and whether the hosts were in one genus or in different genera. The divergence scores were calculated with MEGA 5.0. The neighbor-joining (NJ) analysis^53^ was used to examine the relationships among the taxa and the population samples. The NJ analyses were conducted with 1000 bootstrap replicates.

### Estimate of divergence time

To evaluate the evolution of the host associations within *Cinara* in the mountains of southwest China, we combined other *Cinara* samples from Europe and North America^16^ (Table S4) with our data. We used a Bayesian framework based on the coalescent method to estimate the divergence times in BEAST v.1.7.5^54^,^55^. The site model that was selected was the GTR + I + G by jModeltest. For each species, the sequences of all haplotypes were used in the analysis. The analysis was conducted with an expansion growth model and an uncorrelated lognormal relaxed clock^56^, with a proposed insect molecular clock (COI substitution rate = 1.77% per million years)^57^. The default priors were used. The chains were analyzed for 101.597 million generations, with sampling every 1000 generations. The Tracer v.1.5.0^58^ was used to verify the posterior distribution and the effective sample sizes (ESSs) from the MCMC output. We used the TreeAnnotator v1.7.5 in the BEAST package^55^ to summarize the tree data with “mean height,” and we discarded the first 25% of the trees as the “burn-in” period, which ended long after the stationary chain likelihood values were established.

### Diversification rates

The diversification rate change over time within *Cinara* in the mountains of southwest China was explored based on R applications. Firstly, we used a maximum likelihood method as implemented in LASER v 2.4–1^59^ to determine whether diversification rates of *Cinara* have changed over time. The test statistic for diversification rate-constancy was calculated as: ΔAIC_RC_ = AIC_RC_ − AIC_RV_, where AIC_RC_ was the AIC score for the best fitting rate-constant diversification model, and AIC_RV_ was the AIC for the best fitting rate-variable diversification model. A positive value for ΔAIC_RC_ indicates that the data are best explained by a rate-variable model of diversification. Two rate-constant (pure birth and birth–death model) and three rate-flexible diversification models (a logistic density-dependent speciation rate, an exponential density-dependent and a yule2rate model) were evaluated in our study. Secondly, the Relative Cladogenesis (RC) test was performed in GEIGER v 2.01^60^ to detect shifts of diversification rates within *Cinara*, using the MCC chronogram generated from BEAST analyses. Bonferroni correction was used to adjust the P-values.

## Additional Information

**How to cite this article**: Chen, R. *et al*. DNA barcoding reveals a mysterious high species diversity of conifer-feeding aphids in the mountains of southwest China. *Sci. Rep*. **6**, 20123; doi: 10.1038/srep20123 (2016).

## Supplementary Material

Supplementary Information

## Figures and Tables

**Table 1:** Analysis of intra- and interspecific divergence along different altitudinal gradients among the congeneric species in *Cinara*.

**Altitude (m)/The number of individuals and species**	**Analysis of average intra- and inter-specific divergences of congeneric species in** ***Cinara***	
	**Average inter-specific distance**	**Average intra-specific distance**
1500–2000/20, 11	0.0760 ± 0.0237	0.0015 ± 0.0015
2000–2500/43, 15	0.0841 ± 0.0241	0.0005 ± 0.0012
2500–3000/80, 44	0.1029 ± 0.0217	0.0025 ± 0.0027
3000–3500/77, 39	0.0996 ± 0.0222	0.0014 ± 0.0023
3500–4000/50, 32	0.1082 ± 0.0243	0.0015 ± 0.0015
above 4000/34, 17	0.1062 ± 0.0212	0.0021 ± 0.0023

**Table 2:** Analysis of intra- and interspecific divergence among different environmental variables among the congeneric species in *Cinara*.

**Environment variables**	**Analysis of average intra- and inter-specific divergences of congeneric species in** ***Cinara***	
	**Average inter-specific distance**	**Average intra-specific distance**
Allopatric; same feeding site; hosts in one genus	0.0574 ± 0.0215	0.0091 ± 0.0034
Allopatric; same feeding site; hosts in different genera	0.0741 ± 0.0219	0.0103 ± 0.0021
Allopatric; different feeding site; hosts in one genus	0.1271 ± 0.0171	—
Allopatric; different feeding site; hosts in different genera	0.1352 ± 0.0203	—
Sympatric; same feeding site; hosts in one genus	0.0597 ± 0.0255	0.0015 ± 0.0007
Sympatric; same feeding site; hosts in different genera	0.0891 ± 0.0272	0.0018 ± 0.0011
Sympatric; different feeding site; hosts in one genus	0.1089 ± 0.0233	—
Sympatric; different feeding site; hosts in different genera	0.1243 ± 0.0288	—
