# Supplementary material for: DNA barcoding reveals a mysterious high species diversity of conifer-feeding aphids in the mountains of southwest China
Source: Sci Rep. 2016 Feb 3;6:20123. doi: 10.1038/srep20123 (PMC4738281; doi:10.1038/srep20123)

DNA barcoding reveals a mysterious high species diversity of conifer-feeding aphids in the mountains of southwest China

Rui Chen [1, 2], Li-Yun Jiang[1], Chen Jing[1], Ge-Xia Qiao[1]*

[1] Key Laboratory of Zoological Systematics and Evolution, Institute of Zoology, Chinese Academy of Sciences, No. 1 Beichen West Road, Chaoyang District, Beijing 100101, P.R. China

[2] College of Life Sciences, University of Chinese Academy of Sciences, No. 19, Yuquan Road, Shijingshan District, Beijing 100049, P.R.China

***Corresponding author**: *Ge-Xia Qiao*

Address: Key Laboratory of Zoological Systematics and Evolution, Institute of Zoology, Chinese Academy of Sciences, No. 1 Beichen West Road, Chaoyang District, Beijing 100101, P.R. China.

Phone: +86 10 64807133

E-mail: qiaogx@ioz.ac.cn

Table S1. Species list of *Cinara* in the mountains of southwest China. Red font represents the species we did not find in this study

Species	Host plant family/genera
Cinara alba Zhang	*Picea*
Cinara atrotibialis David *et* Rajasingh	*Pinus*
Cinara brevisaeta Zhang, Zhang et Zhong	*Pinus*
Cinara confinis (Koch, 1956)	*Abies*
Cinara costata (Zetterstedt)	*Picea*
Cinara formosana (Takahashi)	*Pinus*
Cinara largirostris Zhang, Zhang et Zhong	*Pinus*
Cinara laricis (Hartig)	*Larix*
Cinara orientalis (Takahashi)	*Pinus*
Cinara piceae (Panzer)	*Picea*
Cinara pilicornis (Hartig)	*Picea*
Cinara pinea (Mordvilko)	*Pinus*
Cinara pinidensiflorae (Essig et Kuwana)	*Pinus*
Cinara pinihabitans (Mordvilko)	*Pinus*
Cinara pruiniviridis Zhang, Chen, Zhong et Li	*Abies/Picea*
Cinara tujafilina (del Guercio)	Cupressaceae
Cinara subapicula Zhang	*Pinus*
Cinara tibetapini Zhang	*Pinus*

Table S2. The detailed collection information and GenBank accession numbers of *Cinara* species in the mountains of southwest China

No.voucher specimens	Host plants	Feeding sites	Collection date	Elevation	Species	No. candidate species	GB accession numbers/*COI*
CHR001	*Picea* sp.	twig	Jul.1, 2002	3450	*Cinara costata* (Zetterstedt)	1	KP339514
CHR002	*Picea* sp.	branch	Jul.1, 2002	3020	*Cinara* sp.	2	KP339515
CHR003	*Pinus tabuliformis* Carr.	twig	Jul.2, 2002	3020	*Cinara atrotibialis* David *et* Rajasingh	3	KP339516
CHR004	*Larix* sp.	branch	Jul.5, 2002	3450	*Cinara laricis* (Hartig)	4	KP339517
CHR005	*Pinus armandi* Franch.	branch	Aug.20, 2003	2100	*Cinara piniarmandicola* Zhang, Zhang & Zhong	5	KP339518
CHR006	*Pinus yunnanensis* Franch.	twig	Oct.9, 2003	1948	*Cinara pinea* (Mordvilko)	6	KP339519
CHR007	*Platycladus orientalis* (L.) Franco	twig	Aug.5, 2003	3002	*Cinara* sp.	7	KP339520
CHR008	*Picea* sp.	twig	Aug.23, 2003	3290	*Cinara* sp.	8	KP339521
CHR009	*Platycladus orientalis* (L.) Franco	twig	Aug.21, 2003	3060	*Cinara tujafilina* (del Guercio)	9	KP339522
CHR010	*Pinus tabuliformis* Carr.	twig	Aug.21, 2003	3060	*Cinara atrotibialis* David *et* Rajasingh	3	KP339523
CHR011	*Pinus tabuliformis* Carr.	twig	Aug.23, 2003	3700	*Cinara atrotibialis* David *et* Rajasingh	3	KP339524
CHR012	*Pinus tabuliformis* Carr.	twig	Aug.24, 2003	3700	*Cinara atrotibialis* David *et* Rajasingh	3	KP339525
CHR013	*Picea* sp.	twig	Aug.8, 2004	1837	*Cinara pilicornis* (Hartig)	10	KP339526
CHR014	*Larix* sp.	branch	Aug.9, 2004	2053	*Cinara cuneomaculata* (del Guercio)	11	KP339527

CHR015	*Tsuga* sp.	twig	Jul.27, 2005	2230	*Cinara* sp.	12	KP339528
CHR016	*Pinus yunnanensis* Franch.	twig	Apr.7, 2005	2000	*Cinara* sp.	13	KP339529
CHR017	*Pinus armandi* Franch.	branch	May.1, 2005	1710	*Cinara piniarmandicola* Zhang, Zhang & Zhong	5	KP339530
CHR018	*Pinus armandi* Franch.	branch	May.5, 2005	2300	*Cinara piniarmandicola* Zhang, Zhang & Zhong	5	KP339531
CHR019	*Pinus yunnanensis* Franch.	twig	May.5, 2005	2300	*Cinara atrotibialis* David *et* Rajasingh	3	KP339532
CHR020	*Platycladus orientalis* (L.) Franco	twig	Apr.20, 2006	1900	*Cinara tujafilina* (del Guercio)	9	KP339533
CHR021	*Pinus yunnanensis* Franch.	twig	Apr.20, 2006	1900	*Cinara pinea* (Mordvilko)	6	KP339534
CHR022	*Pinus yunnanensis* Franch.	twig	Apr.21, 2006	1900	*Cinara atrotibialis* David *et* Rajasingh	3	KP339535
CHR023	*Pinus armandi* Franch.	branch	Apr.25, 2006	2400	*Cinara piniarmandicola* Zhang, Zhang & Zhong	5	KP339536
CHR024	*Pinus yunnanensis* Franch.	twig	Apr.26, 2006	1920	*Cinara atrotibialis* David *et* Rajasingh	3	KP339537
CHR025	*Pinus yunnanensis* Franch.	twig	Apr.27, 2006	2400	*Cinara formosana* (Takahashi)	14	KP339538
CHR026	*Pinus yunnanensis* Franch.	twig	Apr.27, 2006	2400	*Cinara pinea* (Mordvilko)	6	KP339539
CHR027	*Pinus armandi* Franch.	twig	Apr.27, 2006	2400	*Cinara brevisaeta* Zhang, Zhang et Zhong	15	KP339540
CHR028	*Pinus armandi* Franch.	branch	Apr.27, 2006	2400	*Cinara piniarmandicola* Zhang,	5	KP339541

					Zhang & Zhong		
CHR029	*Pinus armandi* Franch.	branch	Apr.28, 2006	2930	*Cinara piniarmandicola* Zhang, Zhang & Zhong	5	KP339542
CHR030	*Picea likiangensis* (Franch) Pritz	twig	Apr.28, 2006	3220	*Cinara alba* Zhang	16	KP339543
CHR031	*Pinus yunnanensis* Franch.	twig	Apr.28, 2006	2740	*Cinara atrotibialis* David *et* Rajasingh	3	KP339544
CHR032	*Picea* sp.	twig	Apr.29, 2006	3130	*Cinara costata* (Zetterstedt)	1	KP339545
CHR033	*Abies* sp.	twig	Apr.29, 2006	3150	*Cinara pruiniviridis* Zhang, Chen, Zhong et Li	17	KP339546
CHR034	*Pinus armandi* Franch.	branch	Apr.29, 2006	3150	*Cinara piniarmandicola* Zhang, Zhang & Zhong	5	KP339547
CHR035	*Picea* sp.	twig	Apr.30, 2006	2730	*Cinara* sp.	2	KP339548
CHR036	Pinaceae	twig	Aug.16, 2005	3650	*Cinara atrotibialis* David *et* Rajasingh	3	KP339549
CHR037	Pinaceae	twig	Sept.6, 2005	3173	*Cinara atrotibialis* David *et* Rajasingh	3	KP339550
CHR038	Cupressaceae	twig	Apr.30, 2006	2710	*Cinara* sp.	18	KP339551
CHR039	*Picea* sp.	branch	May.2, 2006	3570	*Cinara costata* (Zetterstedt)	1	KP339552
CHR040	*Picea* sp.	twig	May.3, 2006	3320	*Cinara* sp.	19	KP339553
CHR041	*Pinus yunnanensis* Franch.	twig	May.4, 2006	3460	*Cinara* sp.	20	KP339554
CHR042	*Platycladus orientalis* (L.) Franco	twig	May.8, 2006	2200	*Cinara* sp.	21	KP339555
CHR043	*Pinus armandi* Franch.	branch	May.9, 2006	2260	*Cinara piniarmandicola* Zhang, Zhang & Zhong	5	KP339556

CHR044	*Pinus yunnanensis* Franch.	twig	May.9, 2006	2350	*Cinara formosana* (Takahashi)	14	KP339557
CHR045	*Platycladus orientalis* (L.) Franco	twig	May.12, 2006	2150	*Cinara* sp.	21	KP339558
CHR046	*Pinus armandi* Franch.	branch	May.16, 2006	2150	*Cinara piniarmandicola* Zhang, Zhang & Zhong	5	KP339559
CHR047	*Pinus armandi* Franch.	branch	May.16, 2006	2060	*Cinara piniarmandicola* Zhang, Zhang & Zhong	5	KP339560
CHR048	*Juniperus formosana* Hayata.	twig	Jul.8, 2009	2705	*Cinara* sp.	7	KP339561
CHR049	*Pinus tabuliformis* Carr.	twig	Jul.9, 2009	2537	*Cinara* sp.	22	KP339562
CHR050	*Picea* sp.	twig	May.22, 2009	2703	*Cinara pilicornis* (Hartig)	10	KP339563
CHR051	*Picea* sp.	twig	Jun.4, 2009	3871	*Cinara pilicornis* (Hartig)	10	KP339564
CHR052	Cupressaceae	twig	May.24, 2009	2089	*Cinara* sp.	7	KP339565
CHR053	*Picea wilsonii* Mast	twig	May.26, 2009	2361	*Cinara* sp.	23	KP339566
CHR054	*Platycladus orientalis* (L.) Franco	branch	May.28, 2009	2296	*Cinara tujafilina* (del Guercio)	9	KP339567
CHR055	*Pinus armandi* Franch.	branch	Jun.2, 2009	1603	*Cinara piniarmandicola* Zhang, Zhang & Zhong	5	KP339568
CHR056	*Pinus armandi* Franch.	branch	Jun.3, 2009	1521	*Cinara piniarmandicola* Zhang, Zhang & Zhong	5	KP339569
CHR057	*Picea* sp.	branch	Nov.8, 2009	1714	*Cinara* sp.	24	KP339570
CHR058	Cupressaceae	twig	Nov.9, 2009	1811	*Cinara* sp.	25	KP339571
CHR059	*Picea* sp.	branch	Oct.24, 2010	1900	*Cinara* sp.	24	KP339572
CHR060	Pinaceae	branch	Aug.16, 2010	3668	*Cinara piniarmandicola* Zhang, Zhang & Zhong	5	KP339573

CHR061	Cupressaceae	twig	Aug.12, 2010	4782	*Cinara* sp.	7	KP339574
CHR062	*Pinus armandi* Franch.	twig	May.21, 2012	2025	*Cinara brevisaeta* Zhang, Zhang et Zhong	15	KP339575
CHR063	*Pinus armandi* Franch.	twig	May.21, 2012	2065	*Cinara brevisaeta* Zhang, Zhang et Zhong	15	KP339576
CHR064	*Pinus armandi* Franch.	twig	May.21, 2012	2280	*Cinara brevisaeta* Zhang, Zhang et Zhong	15	KP339577
CHR065	*Pinus armandi* Franch.	branch	May.21, 2012	2280	*Cinara piniarmandicola* Zhang, Zhang & Zhong	5	KP339578
CHR066	*Pinus armandi* Franch.	trunk	May.21, 2012	2280	*Cinara piniarmandicola* Zhang, Zhang & Zhong	5	KP339579
CHR067	*Pinus armandi* Franch.	branch	May.21, 2012	2280	*Cinara piniarmandicola* Zhang, Zhang & Zhong	5	KP339580
CHR068	*Pinus yunnanensis* Franch.	twig	May.22, 2012	1953	*Cinara pinea* (Mordvilko)	6	KP339581
CHR069	*Pinus armandi* Franch.	twig	May.22, 2012	2442	*Cinara brevisaeta* Zhang, Zhang et Zhong	15	KP339582
CHR070	*Pinus armandi* Franch.	trunk	May.22, 2012	2468	*Cinara piniarmandicola* Zhang, Zhang & Zhong	5	KP339583
CHR071	*Pinus armandi* Franch.	twig	May.22, 2012	2468	*Cinara brevisaeta* Zhang, Zhang et Zhong	15	KP339584
CHR072	*Pinus yunnanensis* Franch.	twig	May.22, 2012	2468	*Cinara pinea* (Mordvilko)	6	KP339585
CHR073	*Pinus yunnanensis* Franch.	twig	May.23, 2012	2028	*Cinara pinea* (Mordvilko)	6	KP339586
CHR074	*Pinus yunnanensis*	twig	May.23, 2012	2395	*Cinara atrotibialis* David *et*	3	KP339587

	Franch.				Rajasingh		
CHR075	*Pinus armandi* Franch.	twig	May.24, 2012	2589	*Cinara brevisaeta* Zhang, Zhang et Zhong	15	KP339588
CHR076	*Pinus yunnanensis* Franch.	twig	May.24, 2012	3200	*Cinara* sp.	20	KP339589
CHR077	*Tsuga* sp.	twig	May.25, 2012	2960	*Cinara* sp.	26	KP339590
CHR078	*Pinus yunnanensis* Franch.	twig	May.24, 2012	3200	*Cinara atrotibialis* David *et* Rajasingh	3	KP339591
CHR079	*Abies* sp.	twig	May.24, 2012	2372	*Cinara* sp.	27	KP339592
CHR080	*Abies* sp.	twig	May.24, 2012	3298	*Cinara* sp.	28	KP339593
CHR081	*Abies* sp.	twig	May.25, 2012	2960	*Cinara* sp.	29	KP339594
CHR082	*Picea* sp.	branch	May.25, 2012	3224	*Cinara* sp.	24	KP339595
CHR083	*Picea* sp.	twig	May.25, 2012	3258	*Cinara pruiniviridis* Zhang, Chen, Zhong et Li	17	KP339596
CHR084	*Picea* sp.	twig	May.25, 2012	3258	*Cinara* sp.	2	KP339597
CHR085	*Picea* sp.	twig	May.25, 2012	3258	*Cinara* sp.	30	KP339598
CHR086	*Picea* sp.	twig	May.25, 2012	3258	*Cinara costata* (Zetterstedt)	1	KP339599
CHR087	*Picea* sp.	branch	May.25, 2012	3258	*Cinara* sp.	24	KP339600
CHR088	*Picea* sp.	twig	May.25, 2012	3258	*Cinara costata* (Zetterstedt)	1	KP339601
CHR089	*Picea* sp.	twig	May.24, 2012	2982	*Cinara costata* (Zetterstedt)	1	KP339602
CHR090	*Picea* sp.	twig	May.25, 2012	3092	*Cinara costata* (Zetterstedt)	1	KP339603
CHR091	*Abies* sp.	twig	May.25, 2012	3092	*Cinara* sp.	29	KP339604
CHR092	*Picea* sp.	twig	May.25, 2012	3224	*Cinara pruiniviridis* Zhang, Chen, Zhong et Li	17	KP339605
CHR093	*Pinus yunnanensis* Franch.	twig	May.26, 2012	2806	*Cinara atrotibialis* David *et* Rajasingh	3	KP339606

CHR094	*Picea* sp.	twig	May.26, 2012	3137	*Cinara costata* (Zetterstedt)	1	KP339607
CHR095	*Picea* sp.	branch	May.26, 2012	3137	*Cinara* sp.	24	KP339608
CHR096	*Picea* sp.	twig	May.26, 2012	3137	*Cinara pruiniviridis* Zhang, Chen, Zhong et Li	17	KP339609
CHR097	*Platycladus orientalis* (L.) Franco	twig	May.26, 2012	2718	*Cinara tujafilina* (del Guercio)	9	KP339610
CHR098	*Juniperus formosana* Hayata.	twig	May.27, 2012	2718	*Cinara* sp.	7	KP339611
CHR099	*Juniperus formosana* Hayata.	twig	May.27, 2012	2701	*Cinara* sp.	18	KP339612
CHR100	*Pinus yunnanensis* Franch.	twig	May.27, 2012	2701	*Cinara pinea* (Mordvilko)	6	KP339613
CHR101	*Pinus yunnanensis* Franch.	twig	May.27, 2012	2786	*Cinara atrotibialis* David *et* Rajasingh	3	KP339614
CHR102	*Picea* sp.	trunk	May.27, 2012	2712	*Cinara* sp.	24	KP339615
CHR103	*Picea* sp.	branch	May.27, 2012	2712	*Cinara* sp.	31	KP339616
CHR104	*Pinus yunnanensis* Franch.	twig	May.27, 2012	3106	*Cinara* sp.	32	KP339617
CHR105	*Picea* sp.	twig	May.27, 2012	3106	*Cinara pruiniviridis* Zhang, Chen, Zhong et Li	17	KP339618
CHR106	*Picea* sp.	twig	May.27, 2012	3106	*Cinara costata* (Zetterstedt)	1	KP339619
CHR107	*Platycladus orientalis* (L.) Franco	twig	May.26, 2012	2719	*Cinara* sp.	21	KP339620
CHR108	*Pinus yunnanensis* Franch.	twig	May.27, 2012	2949	*Cinara atrotibialis* David *et* Rajasingh	3	KP339621
CHR109	*Picea likiangensis*	twig	May.28, 2012	2787	*Cinara pruiniviridis* Zhang,	17	KP339622

	(Franch) Pritz				Chen, Zhong et Li		
CHR110	*Picea likiangensis* (Franch) Pritz	twig	May.28, 2012	2787	*Cinara* sp.	2	KP339623
CHR111	*Abies* sp.	twig	May.28, 2012	2787	*Cinara* sp.	33	KP339624
CHR112	*Picea* sp.	twig	May.28, 2012	3282	*Cinara pruiniviridis* Zhang, Chen, Zhong et Li	17	KP339625
CHR113	*Picea* sp.	twig	May.28, 2012	3282	*Cinara costata* (Zetterstedt)	1	KP339626
CHR114	*Pinus yunnanensis* Franch.	twig	May.28, 2012	3195	*Cinara pinea* (Mordvilko)	6	KP339627
CHR115	*Pinus yunnanensis* Franch.	trunk	May.29, 2012	3195	*Cinara largirostris* Zhang, Zhang et Zhong	34	KP339628
CHR116	*Picea* sp.	trunk	May.29, 2012	3195	*Cinara* sp.	24	KP339629
CHR117	*Abies* sp.	twig	May.29, 2012	3267	*Cinara* sp.	35	KP339630
CHR118	*Picea* sp.	twig	May.29, 2012	3267	*Cinara* sp.	36	KP339631
CHR119	*Picea* sp.	twig	May.29, 2012	3267	*Cinara costata* (Zetterstedt)	1	KP339632
CHR120	*Larix* sp.	branch	May.29, 2012	3272	*Cinara* sp.	37	KP339633
CHR121	*Juniperus formosana* Hayata.	twig	May.29, 2012	3613	*Cinara* sp.	38	KP339634
CHR122	*Juniperus formosana* Hayata.	twig	May.29, 2012	3613	*Cinara* sp.	39	KP339635
CHR123	*Picea likiangensis* (Franch) Pritz	twig	May.28, 2012	2786	*Cinara costata* (Zetterstedt)	1	KP339636
CHR124	*Picea likiangensis* (Franch) Pritz	twig	May.28, 2012	3309	*Cinara* sp.	2	KP339637
CHR125	*Picea* sp.	twig	May.28, 2012	3282	*Cinara costata* (Zetterstedt)	1	KP339638
CHR126	*Picea likiangensis*	twig	May.30, 2012	2911	*Cinara* sp.	40	KP339639

	(Franch) Pritz						
CHR127	*Tsuga* sp.	twig	May.30, 2012	2832	*Cinara* sp.	41	KP339640
CHR128	*Tsuga* sp.	twig	May.30, 2012	2832	*Cinara* sp.	42	KP339641
CHR129	*Pinus yunnanensis* Franch.	twig	May.30, 2012	2582	*Cinara atrotibialis* David *et* Rajasingh	3	KP339642
CHR130	*Picea likiangensis* (Franch) Pritz	twig	May.30, 2012	2910	*Cinara* sp.	43	KP339643
CHR131	*Picea likiangensis* (Franch) Pritz	branch	May.30, 2012	2910	*Cinara* sp.	36	KP339644
CHR132	*Picea likiangensis* (Franch) Pritz	twig	May.30, 2012	2910	*Cinara* sp.	44	KP339645
CHR133	*Picea likiangensis* (Franch) Pritz	twig	May.30, 2012	2910	*Cinara costata* (Zetterstedt)	1	KP339646
CHR134	*Pinus yunnanensis* Franch.	branch	May.30, 2012	2832	*Cinara largirostris* Zhang, Zhang et Zhong	34	KP339647
CHR135	*Pinus yunnanensis* Franch.	twig	May.31, 2012	2832	*Cinara atrotibialis* David *et* Rajasingh	3	KP339648
CHR136	*Pinus yunnanensis* Franch.	twig	May.31, 2012	2461	*Cinara pinea* (Mordvilko)	6	KP339649
CHR137	*Pinus yunnanensis* Franch.	branch	May.31, 2012	2461	*Cinara largirostris* Zhang, Zhang et Zhong	34	KP339650
CHR138	*Abies* sp.	twig	May.31, 2012	2732	*Cinara* sp.	45	KP339651
CHR139	*Pinus yunnanensis* Franch.	twig	May.31, 2012	1875	*Cinara pinea* (Mordvilko)	6	KP339652
CHR140	*Tsuga* sp.	twig	Jun.1, 2012	3264	*Cinara* sp.	46	KP339653
CHR141	*Tsuga* sp.	twig	Jun.1, 2012	3264	*Cinara* sp.	41	KP339654

CHR142	*Pinus armandi* Franch.	branch	Jun.1, 2012	3264	*Cinara piniarmandicola* Zhang, Zhang & Zhong	5	KP339655
CHR143	*Abies delavayi* Franch.	twig	Jun.1, 2012	3264	*Cinara* sp.	47	KP339656
CHR144	*Abies delavayi* Franch.	twig	Jun.1, 2012	3264	*Cinara* sp.	48	KP339657
CHR145	*Abies* sp.	twig	Jun.1, 2012	3260	*Cinara* sp.	49	KP339658
CHR146	*Pinus yunnanensis* Franch.	twig	Jun.1, 2012	2019	*Cinara atrotibialis* David *et* Rajasingh	3	KP339659
CHR147	*Pinus yunnanensis* Franch.	twig	Jun.2, 2012	2019	*Cinara pinea* (Mordvilko)	6	KP339660
CHR148	*Picea* sp.	twig	Jun.20, 2012	2620	*Cinara* sp.	50	KP339661
CHR149	*Abies* sp.	twig	Jun.20, 2012	2844	*Cinara* sp.	51	KP339662
CHR150	*Abies* sp.	twig	Jun.20, 2012	2844	*Cinara* sp.	52	KP339663
CHR151	*Abies* sp.	twig	Jun.20, 2012	2844	*Cinara* sp.	51	KP339664
CHR152	*Picea* sp.	twig	Jun.20, 2012	2844	*Cinara* sp.	50	KP339665
CHR153	*Picea wilsonii* Mast	twig	Jun.20, 2012	2844	*Cinara* sp.	53	KP339666
CHR154	*Abies* sp.	twig	Jun.20, 2012	2842	*Cinara* sp.	54	KP339667
CHR155	*Picea* sp.	twig	Jun.20, 2012	2842	*Cinara* sp.	55	KP339668
CHR156	*Pinus tabuliformis* Carr.	twig	Jun.21, 2012	2376	*Cinara* sp.	22	KP339669
CHR157	*Larix* sp.	branch	Jun.21, 2012	2844	*Cinara cuneomaculata* (del Guercio)	11	KP339670
CHR158	*Pinus armandi* Franch.	branch	Jun.21, 2012	2844	*Cinara piniarmandicola* Zhang, Zhang & Zhong	5	KP339671
CHR159	*Abies* sp.	twig	Jun.22, 2012	2961	*Cinara* sp.	56	KP339672
CHR160	*Picea* sp.	twig	Jun.22, 2012	2961	*Cinara* sp.	50	KP339673
CHR161	*Picea* sp.	twig	Jun.22, 2012	2754	*Cinara* sp.	23	KP339674

CHR162	*Picea* sp.	twig	Jun.22, 2012	2754	*Cinara* sp.	23	KP339675
CHR163	*Picea* sp.	twig	Jun.23, 2012	2611	*Cinara* sp.	50	KP339676
CHR164	*Platycladus orientalis* (L.) Franco	twig	Jun.26, 2012	1500	*Cinara tujafilina* (del Guercio)	9	KP339677
CHR165	*Pinus tabuliformis* Carr.	twig	Jun.26, 2012	1670	*Cinara* sp.	22	KP339678
CHR166	*Pinus tabuliformis* Carr.	twig	Jun.27, 2012	1731	*Cinara* sp.	22	KP339679
CHR167	*Larix* sp.	branch	Jun.27, 2012	1731	*Cinara cuneomaculata* (del Guercio)	11	KP339680
CHR168	*Picea* sp.	twig	Jun.27, 2012	2054	*Cinara* sp.	23	KP339681
CHR169	*Pinus armandi* Franch.	twig	Jun.27, 2012	1731	*Cinara* sp.	57	KP339682
CHR170	*Picea* sp.	branch	Aug.9, 2012	4082	*Cinara* sp.	24	KP339683
CHR171	*Picea* sp.	twig	Aug.9, 2012	4082	*Cinara* sp.	58	KP339684
CHR172	*Abies* sp.	twig	Jul.26, 2012	3419	*Cinara* sp.	59	KP339685
CHR173	*Abies* sp.	twig	Jul.26, 2012	3419	*Cinara* sp.	8	KP339686
CHR174	*Picea* sp.	twig	Jul.18, 2012	4230	*Cinara* sp.	60	KP339687
CHR175	*Picea* sp.	twig	Jul.18, 2012	4230	*Cinara* sp.	61	KP339688
CHR176	*Juniperus formosana* Hayata.	twig	Jul.22, 2012	4069	*Cinara* sp.	62	KP339689
CHR177	Cupressaceae	twig	Aug.13, 2012	3747	*Cinara* sp.	63	KP339690
CHR178	*Picea* sp.	twig	Aug.13, 2012	3747	*Cinara* sp.	64	KP339691
CHR179	*Abies* sp.	twig	Aug.2, 2012	3419	*Cinara* sp.	65	KP339692
CHR180	*Abies* sp.	twig	Aug.2, 2012	3419	*Cinara* sp.	65	KP339693
CHR181	*Larix* sp.	branch	Jul.11, 2012	2977	*Cinara* sp.	66	KP339694
CHR182	*Pinus tabuliformis* Carr.	branch	Jul.25, 2012	2461	*Cinara largirostris* Zhang, Zhang et Zhong	34	KP339695

CHR183	*Abies* sp.	twig	Jul.11, 2012	2977	*Cinara* sp.	67	KP339696
CHR184	*Abies* sp.	twig	Jul.9, 2012	3340	*Cinara* sp.	67	KP339697
CHR185	*Abies* sp.	twig	Jul.9, 2012	3340	*Cinara* sp.	68	KP339698
CHR186	*Abies* sp.	twig	Jul.9, 2012	3340	*Cinara* sp.	67	KP339699
CHR187	*Abies* sp.	twig	Jul.24, 2012	4571	*Cinara* sp.	69	KP339700
CHR188	*Abies* sp.	twig	Jul.24, 2012	4571	*Cinara* sp.	69	KP339701
CHR189	*Picea* sp.	twig	Aug.12, 2012	4668	*Cinara* sp.	70	KP339702
CHR190	*Picea* sp.	twig	Aug.12, 2012	4668	*Cinara* sp.	61	KP339703
CHR191	*Picea* sp.	branch	Jul.14, 2012	3369	*Cinara* sp.	24	KP339704
CHR192	*Picea brachytyla* (Franch.)	twig	Aug.5, 2013	2595	*Cinara* sp.	71	KP339705
CHR193	*Picea brachytyla* (Franch.)	twig	Aug.5, 2013	2595	*Cinara* sp.	2	KP339706
CHR194	*Picea brachytyla* (Franch.)	twig	Aug.5, 2013	2595	*Cinara* sp.	72	KP339707
CHR195	*Picea brachytyla* (Franch.)	trunk	Aug.5, 2013	2595	*Cinara* sp.	24	KP339708
CHR196	*Picea brachytyla* (Franch.)	twig	Aug.5, 2013	2860	*Cinara* sp.	72	KP339709
CHR197	*Larix* sp.	branch	Aug.5, 2013	2860	*Cinara* sp.	66	KP339710
CHR198	*Picea brachytyla* (Franch.)	twig	Aug.5, 2013	2860	*Cinara* sp.	2	KP339711
CHR199	*Tsuga* sp.	twig	Aug.5, 2013	2860	*Cinara* sp.	73	KP339712
CHR200	*Larix* sp.	branch	Aug.6, 2013	3086	*Cinara* sp.	66	KP339713
CHR201	*Picea* sp.	twig	Aug.6, 2013	3086	*Cinara pilicornis* (Hartig)	10	KP339714
CHR202	*Picea* sp.	twig	Aug.6, 2013	3086	*Cinara pilicornis* (Hartig)	10	KP339715

CHR203	*Picea* sp.	twig	Aug.6, 2013	3086	*Cinara* sp.	2	KP339716
CHR204	*Picea brachytyla* (Franch.)	twig	Aug.6, 2013	2690	*Cinara pruiniviridis* Zhang, Chen, Zhong et Li	17	KP339717
CHR205	*Pinus armandi* Franch.	branch	Aug.7, 2013	2761	*Cinara piniarmandicola* Zhang, Zhang & Zhong	5	KP339718
CHR206	*Picea* sp.	twig	Aug.7, 2013	2849	*Cinara* sp.	50	KP339719
CHR207	*Picea* sp.	twig	Aug.7, 2013	2849	*Cinara pilicornis* (Hartig)	10	KP339720
CHR208	*Picea* sp.	twig	Aug.7, 2013	2849	*Cinara* sp.	74	KP339721
CHR209	*Picea* sp.	twig	Aug.8, 2013	3880	*Cinara* sp.	75	KP339722
CHR210	*Picea* sp.	twig	Aug.8, 2013	3880	*Cinara* sp.	64	KP339723
CHR211	*Picea* sp.	twig	Aug.8, 2013	3880	*Cinara* sp.	76	KP339724
CHR212	*Picea* sp.	twig	Aug.8, 2013	3880	*Cinara* sp.	77	KP339725
CHR213	*Picea* sp.	branch	Aug.8, 2013	3880	*Cinara* sp.	24	KP339726
CHR214	*Picea* sp.	twig	Aug.8, 2013	3880	*Cinara* sp.	78	KP339727
CHR215	*Larix* sp.	branch	Aug.8, 2013	3369	*Cinara* sp.	79	KP339728
CHR216	*Picea* sp.	twig	Aug.8, 2013	4308	*Cinara* sp.	61	KP339729
CHR217	*Picea* sp.	branch	Aug.9, 2013	3930	*Cinara* sp.	24	KP339730
CHR218	*Picea* sp.	twig	Aug.9, 2013	3930	*Cinara* sp.	64	KP339731
CHR219	*Larix* sp.	branch	Aug.9, 2013	4036	*Cinara* sp.	79	KP339732
CHR220	*Picea* sp.	twig	Aug.9, 2013	4036	*Cinara* sp.	80	KP339733
CHR221	*Picea* sp.	branch	Aug.9, 2013	4036	*Cinara* sp.	24	KP339734
CHR222	*Picea* sp.	twig	Aug.9, 2013	4036	*Cinara* sp.	75	KP339735
CHR223	Cupressaceae	twig	Aug.9, 2013	4036	*Cinara* sp.	62	KP339736
CHR224	*Pinus massoniana* Lamb.	twig	Aug.9, 2013	3612	*Cinara* sp.	81	KP339737

CHR225	*Pinus massoniana* Lamb.	twig	Aug.10, 2013	3160	*Cinara* sp.	81	KP339738
CHR226	*Pinus massoniana* Lamb.	twig	Aug.10, 2013	3160	*Cinara atrotibialis* David *et* Rajasingh	3	KP339739
CHR227	*Pinus massoniana* Lamb.	branch	Aug.10, 2013	3753	*Cinara largirostris* Zhang, Zhang et Zhong	34	KP339740
CHR228	*Larix* sp.	branch	Aug.10, 2013	4106	*Cinara* sp.	37	KP339741
CHR229	*Abies* sp.	twig	Aug.10, 2013	4333	*Cinara* sp.	51	KP339742
CHR230	*Abies* sp.	twig	Aug.10, 2013	4333	*Cinara* sp.	51	KP339743
CHR231	*Abies* sp.	twig	Aug.10, 2013	4333	*Cinara* sp.	51	KP339744
CHR232	Cupressaceae	twig	Aug.10, 2013	4188	*Cinara* sp.	62	KP339745
CHR233	*Pinus armandi* Franch.	trunk	Aug.10, 2013	3029	*Cinara piniarmandicola* Zhang, Zhang & Zhong	5	KP339746
CHR234	Cupressaceae	twig	Aug.11, 2013	3180	*Cinara tujafilina* (del Guercio)	9	KP339747
CHR235	*Larix* sp.	branch	Aug.12, 2013	3799	*Cinara* sp.	79	KP339748
CHR236	*Larix* sp.	branch	Aug.12, 2013	3799	*Cinara* sp.	37	KP339749
CHR237	*Picea* sp.	twig	Aug.12, 2013	3933	*Cinara* sp.	75	KP339750
CHR238	*Picea* sp.	twig	Aug.12, 2013	3933	*Cinara* sp.	80	KP339751
CHR239	Cupressaceae	twig	Aug.12, 2013	3933	*Cinara* sp.	62	KP339752
CHR240	*Larix* sp.	branch	Aug.12, 2013	3933	*Cinara* sp.	37	KP339753
CHR241	*Abies* sp.	twig	Aug.12, 2013	3933	*Cinara* sp.	76	KP339754
CHR242	*Picea* sp.	branch	Aug.12, 2013	4153	*Cinara* sp.	24	KP339755
CHR243	*Picea* sp.	twig	Aug.12, 2013	4153	*Cinara* sp.	76	KP339756
CHR244	*Abies* sp.	twig	Aug.12, 2013	3840	*Cinara* sp.	77	KP339757
CHR245	*Picea* sp.	twig	Aug.12, 2013	3840	*Cinara* sp.	23	KP339758

CHR246	*Picea* sp.	twig	Aug.12, 2013	3840	*Cinara* sp.	76	KP339759
CHR247	*Picea* sp.	twig	Aug.12, 2013	3840	*Cinara* sp.	77	KP339760
CHR248	*Picea* sp.	twig	Aug.12, 2013	3840	*Cinara* sp.	77	KP339761
CHR249	*Abies* sp.	twig	Aug.13, 2013	3174	*Cinara* sp.	82	KP339762
CHR250	*Picea* sp.	branch	Aug.13, 2013	3174	*Cinara* sp.	24	KP339763
CHR251	*Picea* sp.	twig	Aug.14, 2013	4035	*Cinara pilicornis* (Hartig)	10	KP339764
CHR252	*Picea* sp.	twig	Aug.14, 2013	4035	*Cinara* sp.	2	KP339765
CHR253	*Picea* sp.	twig	Aug.14, 2013	4035	*Cinara pilicornis* (Hartig)	10	KP339766
CHR254	*Picea* sp.	twig	Aug.14, 2013	4035	*Cinara* sp.	75	KP339767
CHR255	Cupressaceae	twig	Aug.14, 2013	4315	*Cinara* sp.	62	KP339768
CHR256	*Picea* sp.	twig	Aug.15, 2013	4018	*Cinara* sp.	83	KP339769
CHR257	*Picea* sp.	twig	Aug.15, 2013	4018	*Cinara* sp.	75	KP339770
CHR258	*Picea* sp.	branch	Aug.15, 2013	4018	*Cinara* sp.	24	KP339771
CHR259	*Picea* sp.	branch	Aug.15, 2013	4035	*Cinara* sp.	24	KP339772
CHR260	*Picea* sp.	twig	Aug.16, 2013	3421	*Cinara* sp.	50	KP339773
CHR261	*Picea* sp.	twig	Aug.16, 2013	3421	*Cinara* sp.	74	KP339774
CHR262	*Picea* sp.	twig	Aug.16, 2013	3451	*Cinara pilicornis* (Hartig)	10	KP339775
CHR263	*Picea* sp.	twig	Aug.16, 2013	3451	*Cinara* sp.	64	KP339776
CHR264	*Picea* sp.	twig	Aug.16, 2013	3451	*Cinara pilicornis* (Hartig)	10	KP339777
CHR265	*Picea* sp.	twig	Aug.16, 2013	3760	*Cinara* sp.	61	KP339778
CHR266	*Picea* sp.	twig	Aug.16, 2013	3760	*Cinara* sp.	84	KP339779
CHR267	*Picea* sp.	twig	Aug.16, 2013	4178	*Cinara* sp.	2	KP339780
CHR268	*Abies* sp.	twig	Aug.17, 2013	2883	*Cinara* sp.	85	KP339781
CHR269	*Picea* sp.	branch	Aug.17, 2013	2883	*Cinara* sp.	24	KP339782
CHR270	*Picea* sp.	twig	Aug.17, 2013	2917	*Cinara* sp.	50	KP339783

CHR271	*Picea* sp.	twig	Aug.17, 2013	2917	*Cinara* sp.	74	KP339784
CHR272	*Picea* sp.	twig	Aug.17, 2013	2917	*Cinara pilicornis* (Hartig)	10	KP339785
CHR273	*Picea* sp.	twig	Aug.17, 2013	2917	*Cinara pilicornis* (Hartig)	10	KP339786
CHR274	Cupressaceae	twig	Aug.17, 2013	4145	*Cinara* sp.	7	KP339787
CHR275	*Abies* sp.	twig	Aug.18, 2013	3753	*Cinara* sp.	86	KP339788
CHR276	*Picea brachytyla* (Franch.)	twig	Aug.18, 2013	3767	*Cinara* sp.	87	KP339789
CHR277	*Picea brachytyla* (Franch.)	twig	Aug.18, 2013	3767	*Cinara* sp.	87	KP339790
CHR278	*Picea* sp.	twig	Aug.18, 2013	3454	*Cinara* sp.	50	KP339791
CHR279	*Picea* sp.	twig	Aug.18, 2013	3454	*Cinara* sp.	74	KP339792
CHR280	*Picea brachytyla* (Franch.)	twig	Aug.18, 2013	3915	*Cinara* sp.	83	KP339793
CHR281	*Picea brachytyla* (Franch.)	twig	Aug.18, 2013	3915	*Cinara* sp.	2	KP339794
CHR282	*Picea brachytyla* (Franch.)	twig	Aug.18, 2013	3915	*Cinara* sp.	88	KP339795
CHR283	*Picea* sp.	twig	Aug.19, 2013	3913	*Cinara* sp.	23	KP339796
CHR284	*Picea* sp.	twig	Aug.19, 2013	3913	*Cinara pilicornis* (Hartig)	10	KP339797
CHR285	*Picea* sp.	twig	Aug.20, 2013	2682	*Cinara* sp.	50	KP339798
CHR286	*Picea* sp.	branch	Aug.20, 2013	2682	*Cinara* sp.	55	KP339799
CHR287	*Picea* sp.	branch	Aug.20, 2013	2682	*Cinara* sp.	89	KP339800
CHR288	Cupressaceae	twig	Aug.20, 2013	2682	*Cinara* sp.	7	KP339801
CHR289	*Abies* sp.	twig	Aug.20, 2013	3522	*Cinara* sp.	90	KP339802
CHR290	*Abies* sp.	twig	Aug.20, 2013	3522	*Cinara* sp.	91	KP339803

CHR291	*Picea* sp.	branch	Aug.20, 2013	3000	*Cinara* sp.	74	KP339804
CHR292	*Picea* sp.	twig	Aug.20, 2013	3000	*Cinara* sp.	50	KP339805
CHR293	Cupressaceae	branch	Aug.20, 2013	3000	*Cinara* sp.	7	KP339806
CHR294	*Larix* sp.	branch	Aug.20, 2013	2897	*Cinara* sp.	92	KP339807
CHR295	Cupressaceae	twig	Aug.20, 2013	2328	*Cinara* sp.	7	KP339808
CHR296	*Picea* sp.	twig	Aug.21, 2013	2313	*Cinara pilicornis* (Hartig)	10	KP339809
CHR297	*Pinus armandi* Franch.	branch	Aug.21, 2013	2313	*Cinara piniarmandicola* Zhang, Zhang & Zhong	5	KP339810
CHR298	*Picea* sp.	twig	Aug.22, 2013	3524	*Cinara* sp.	50	KP339811
CHR299	*Picea brachytyla* (Franch.)	twig	Aug.22, 2013	3524	*Cinara* sp.	93	KP339812
CHR300	*Picea brachytyla* (Franch.)	twig	Aug.22, 2013	3524	*Cinara* sp.	23	KP339813
CHR301	*Picea brachytyla* (Franch.)	twig	Aug.22, 2013	3524	*Cinara* sp.	93	KP339814
CHR302	*Abies* sp.	twig	Aug.22, 2013	3524	*Cinara* sp.	94	KP339815
CHR303	*Abies* sp.	twig	Aug.22, 2013	3524	*Cinara* sp.	91	KP339816
CHR304	*Pinus armandi* Franch.	branch	Aug.23, 2013	2335	*Cinara piniarmandicola* Zhang, Zhang & Zhong	5	KP339817

Table S3. Altitudinal range of *Cinara* species/candidate species in the mountains of southwest China

Number of species/candidate species	Altitude range (m)
1	2500-4500
2	2500-4500
3	1500-4000
4	3000-4000
5	1200-3800
6	1500-4000
7	2000-5000
8	3000-4000
9	0-3500
10	1500-4200
11	1500-4000
12	2000-3000
13	1500-4000
14	1500-4000
15	2000-3000
16	3000-4000
17	2500-4000
18	2000-3000
19	3000-4000
20	2800-4000
21	2000-3000
22	1000-3000
23	2000-4000
24	1500-4200
25	1500-2500
26	2500-3500
27	2000-3500
28	2000-3500
29	2000-3500
30	2000-3500
31	2000-3500
32	2000-3500
33	2000-3500
34	2000-4000
35	2500-3500
36	2500-3500
37	2500-4500
38	3000-4200
39	3000-4200
40	2500-4000

41	2500-3500
42	2500-3500
44	2500-4000
45	2500-4000
46	2500-3500
46	2500-4000
47	2500-4000
48	2500-4000
49	2500-4000
50	2500-4000
51	2500-4800
52	2500-4000
53	2500-4000
54	2500-4000
55	2500-4000
56	2500-4000
57	1200-3800
58	3000-4500
59	3000-4000
60	3500-5000
61	3000-5000
62	3500-4800
63	3000-4200
64	3000-4200
65	3000-4200
66	2500-4000
67	2500-4000
68	2500-4000
69	4000-5000
70	4000-5000
71	2000-3500
72	2000-3500
73	2000-3500
74	2500-4000
75	3200-4500
76	3200-4500
77	3200-4500
78	3200-4500
79	3000-4500
80	3200-4500
81	2500-4000
82	2500-4200
83	3200-4500

84	3200-4500
85	2500-4200
86	3000-4200
87	3000-4200
88	3000-4200
89	2000-4000
90	3000-4500
91	3000-4500
92	2000-4000
93	3000-4200
94	3000-4200

Table S4. The species information and GenBank accession numbers of *Cinara* downloaded from GenBank Fr:France, US: United States of America, KZ: Kazakhstan,It: Italy, Gr:Greece, Al: Algeria (Jousselin et al. 2013)

Voucher	Genus	Species	Host genus	Location	Feeding site recorded in the field	COI
2956	*Cinara*	*anelia*	*Pinus*	California (US)	branch	KF649467
2961	*Cinara*	*anelia*	*Pinus*	California (US)	branch	KF649469
2967	*Cinara*	*anelia*	*Pinus*	California (US)	branch	KF649472
2920	*Cinara*	*apini*	*Pinus*	New-Mexico (US)	branch	KF649451
2865	*Cinara*	*apini*	*Pinus*	Colorado (US)	branch	KF649403
2995	*Cinara*	*arizonica*	*Pinus*	California (US)	branch	KF649487
3044	*Cinara*	*arizonica*	*Pinus*	Oregon (US)	branch	KF649514
463	*Cinara*	*brauni*	*Pinus*	Languedoc-Roussillon (Fr)	shoot	KF649558
2827	*Cinara*	*brauni*	*Pinus*	Almaty (KZ)	shoot	KF649394
2988	*Cinara*	*brevispinosa*	*Pinus*	California (US)	unknown	KF649484
3016	*Cinara*	*brevispinosa*	*Pinus*	Washington (US)	shoot	KF649499
3040	*Cinara*	*brevispinosa*	*Pinus*	Washington (US)	shoot	KF649513
3060	*Cinara*	*brevispinosa*	*Pinus*	Oregon (US)	shoot	KF649525
1971	*Cinara*	*cedri*	*Cedrus*	Sicily (It)	shoot	KF649349
1973	*Cinara*	*cedri*	*Cedrus*	Sicily (It)	shoot	KF649350
1974	*Cinara*	*cedri*	*Cedrus*	Sicily (It)	shoot	KF649351
2802	*Cinara*	*cedri*	*Cedrus*	Languedoc-Roussillon (Fr)	branch	KF649387
2834	*Cinara*	*cedri*	*Cedrus*	Languedoc-Roussillon (Fr)	branch	KF649397
3033	*Cinara*	*cedri*	*Cedrus*	Washington (US)	branch	KF649509
2706	*Cinara*	*cembrae*	*Pinus*	PACA (Fr)	branch	KF649368
2760	*Cinara*	*cembrae*	*Pinus*	PACA (Fr)	branch	KF649381
2879	*Cinara*	*close_C_ piceae*	*Picea*	Colorado (US)	branch	KF649413

2880	*Cinara*	*close_C_ piceae*	*Picea*	Colorado (US)	branch	KF649414
2914	*Cinara*	*close_C_ piceae*	*Picea*	New-Mexico (US)	branch	KF649445
2972	*Cinara*	*close_C_ piceae*	*Picea*	California (US)	branch	KF649476
2903a	*Cinara*	*close_C_ piceae*	*Picea*	Colorado (US)	branch	KF649434
3048	*Cinara*	*close_coloradensis*	*Picea*	Oregon (US)	branch	KF649517
2466	*Cinara*	*confinis*	*Abies*	Burgundy (Fr)	branch	KF649359
2800	*Cinara*	*confinis*	*Abies*	Languedoc-Roussillon (Fr)	trunk	KF649385
2801	*Cinara*	*confinis*	*Abies*	Languedoc-Roussillon (Fr)	branch	KF649386
3010	*Cinara*	*contortae*	*Pinus*	Oregon (US)	branch	KF649497
3034	*Cinara*	*contortae*	*Pinus*	Washington (US)	shoot	KF649510
3050	*Cinara*	*contortae*	*Pinus*	Oregon (US)	branch	KF649519
2612	*Cinara*	*cuneomaculata*	*Larix*	Rhone-Alpes (Fr)	branch	KF649360
2692	*Cinara*	*cuneomaculata*	*Larix*	PACA (Fr)	branch	KF649365
2693	*Cinara*	*cuneomaculata*	*Larix*	PACA (Fr)	branch	KF649366
2700	*Cinara*	*cuneomaculata*	*Larix*	PACA (Fr)	branch	KF649367
445a	*Cinara*	*cuneomaculata*	*Larix*	Languedoc-Roussillon (Fr)	unknown	KF649555
3053	*Cinara*	*curtihirsuta*	*Abies*	Oregon (US)	trunk	KF649522
3070	*Cinara*	*curtihirsuta*	*Abies*	Oregon (US)	branch	KF649533
3072	*Cinara*	*curtihirsuta*	*Abies*	California (US)	branch	KF649535
3075	*Cinara*	*curtihirsuta*	*Abies*	California (US)	branch	KF649538
3078	*Cinara*	*curtihirsuta*	*Abies*	California (US)	branch	KF649541
2891	*Cinara*	*edulis*	*Pinus*	Colorado (US)	branch	KF649424
2894	*Cinara*	*edulis*	*Pinus*	Colorado (US)	branch	KF649427
2904	*Cinara*	*englemanniensis_bonica*	*Picea*	Colorado (US)	branch	KF649436
2907	*Cinara*	*fornacula*	*Picea*	Colorado (US)	shoot	KF649439

2912	*Cinara*	*fornacula*	*Picea*	Colorado (US)	shoot	KF649443
2983	*Cinara*	*fresai*	*Cupressus*	California (US)	shoot	KF649480
2993	*Cinara*	*fresai*	*Cupressus*	California (US)	shoot	KF649486
3003	*Cinara*	*fresai*	*Cupressus*	Oregon (US)	shoot	KF649492
3038	*Cinara*	*fresai*	*Cupressus*	Washington (US)	shoot	KF649511
1785	*Cinara*	*fresai*	*Juniperus*	Haute-Normandie (Fr)	shoot	KF649347
2924a	*Cinara*	*glabra*	*Pinus*	New-Mexico (US)	shoot	KF649455
2969b	*Cinara*	*glabra*	*Pinus*	California (US)	branch	KF649474
2870b	*Cinara*	*glabra*	*Pinus*	Colorado (US)	shoot	KF649406
2906	*Cinara*	*grande*	*Abies*	Colorado (US)	branch	KF649438
2925	*Cinara*	*gudaris*	*Pinus*	Languedoc-Roussillon (Fr)	shoot	KF649458
2903b	*Cinara*	*hottesi*	*Picea*	Colorado (US)	branch	KF649435
2709	*Cinara*	*juniperi*	*Juniperus*	PACA (Fr)	shoot	KF649370
2720	*Cinara*	*juniperi*	*Juniperus*	PACA (Fr)	shoot	KF649372
2763	*Cinara*	*juniperi*	*Juniperus*	PACA (Fr)	shoot	KF649383
2952	*Cinara*	*juniperi*	*Juniperus*	PACA (Fr)	unknown	KF649465
640bis	*Cinara*	*juniperi*	*Juniperus*	Aquitaine (Fr)	unknown	KF649559
2762	*Cinara*	*kochiana*	*Larix*	PACA (Fr)	trunk	KF649382
2940	*Cinara*	*kochiana*	*Larix*	PACA (Fr)	trunk	KF649462
3058	*Cinara*	*laricifoliae*	*Larix*	Oregon (US)	branch	KF649524
3068	*Cinara*	*laricifoliae*	*Larix*	Oregon (US)	branch	KF649532
2708	*Cinara*	*laricis*	*Larix*	PACA (Fr)	branch	KF649369
2945	*Cinara*	*laricis*	*Larix*	PACA (Fr)	branch	KF649464
3094	*Cinara*	*laricis*	*Larix*	Valais (CH)	branch	KF649554
445b	*Cinara*	*laricis*	*Larix*	Languedoc-Roussillon (Fr)	unknown	KF649556

2382	*Cinara*	*maghrebica*	*Pinus*	Khenchela (Al)	unknown	KF649357
2873	*Cinara*	*medispinosa*	*Pinus*	Colorado (US)	shoot	KF649409
2874	*Cinara*	*medispinosa*	*Pinus*	Colorado (US)	shoot	KF649410
2875	*Cinara*	*medispinosa*	*Pinus*	Colorado (US)	branch	KF649411
3080	*Cinara*	*moketa*	*Pinus*	California (US)	branch	KF649543
3084	*Cinara*	*moketa*	*Pinus*	California (US)	branch	KF649547
3085	*Cinara*	*moketa*	*Pinus*	California (US)	branch	KF649548
3086	*Cinara*	*moketa*	*Pinus*	California (US)	branch	KF649549
3087	*Cinara*	*moketa*	*Pinus*	California (US)	branch	KF649550
3051	*Cinara*	*murrayanae*	*Pinus*	Oregon (US)	branch	KF649520
3063	*Cinara*	*murrayanae*	*Pinus*	Oregon (US)	branch	KF649528
3066	*Cinara*	*murrayanae*	*Pinus*	Oregon (US)	branch	KF649530
3067	*Cinara*	*murrayanae*	*Pinus*	Oregon (US)	shoot	KF649531
3071	*Cinara*	*murrayanae*	*Pinus*	Oregon (US)	branch	KF649534
3065	*Cinara*	*murrayanae_ medispinosa*	*Pinus*	Oregon (US)	gall	KF649529
2829	*Cinara*	*near_guadarramae*	*Pinus*	Almaty (KZ)	branch	KF649395
2747	*Cinara*	*neubergi*	*Pinus*	PACA (Fr)	shoot	KF649380
2837	*Cinara*	*nuda*	*Pinus*	Aquitaine (Fr)	branch	KF649399
2902	*Cinara*	*obscura_sitchensis*	*Picea*	Colorado (US)	branch	KF649433
2910	*Cinara*	*obscura_sitchensis*	*Picea*	Colorado (US)	shoot	KF649441
2911	*Cinara*	*obscura_sitchensis*	*Picea*	Colorado (US)	branch	KF649442
2913b	*Cinara*	*obscura_sitchensis*	*Picea*	Colorado (US)	branch	KF649444
2884	*Cinara*	*occidentalis*	*Abies*	Colorado (US)	shoot	KF649418
3024	*Cinara*	*occidentalis*	*Abies*	Washington (US)	shoot	KF649503
3049	*Cinara*	*occidentalis*	*Abies*	Oregon (US)	branch	KF649518

3074	*Cinara*	*occidentalis*	*Abies*	California (US)	branch	KF649537
3079	*Cinara*	*occidentalis*	*Abies*	California (US)	branch	KF649542
3081	*Cinara*	*occidentalis*	*Abies*	California (US)	branch	KF649544
3090	*Cinara*	*occidentalis*	*Abies*	California (US)	branch	KF649551
2916	*Cinara*	*occidentalis*	*Pseudotsuga*	New-Mexico (US)	branch	KF649447
1046	*Cinara*	*palaestinensis*	*Pinus*	Languedoc-Roussillon (Fr)	unknown	KF649338
1542	*Cinara*	*palaestinensis*	*Pinus*	Peloponèse (Gr)	shoot	KF649346
446	*Cinara*	*pectinatae*	*Abies*	Languedoc-Roussillon (Fr)	unknown	KF649557
2725	*Cinara*	*pectinatae*	*Abies*	Languedoc-Roussillon (Fr)	shoot	KF649375
2727	*Cinara*	*pectinatae*	*Abies*	Languedoc-Roussillon (Fr)	unknown	KF649377
2905	*Cinara*	*petersoni*	*Juniperus*	Colorado (US)	shoot	KF649437
2188	*Cinara*	*pilicornis*	*Picea*	Almaty (KZ)	shoot	KF649354
2674	*Cinara*	*pilicornis*	*Picea*	Languedoc-Roussillon (Fr)	shoot	KF649362
2769	*Cinara*	*pilicornis*	*Picea*	PACA (Fr)	branch	KF649384
2805	*Cinara*	*pilicornis*	*Picea*	Almaty (KZ)	shoot	KF649389
2811	*Cinara*	*pilicornis*	*Picea*	Almaty (KZ)	branch	KF649392
2826	*Cinara*	*pilicornis*	*Picea*	Almaty (KZ)	shoot	KF649393
2984	*Cinara*	*pilicornis*	*Picea*	California (US)	shoot	KF649481
2992	*Cinara*	*pilicornis*	*Picea*	California (US)	shoot	KF649485
3006	*Cinara*	*pilicornis*	*Picea*	Oregon (US)	shoot	KF649495
3023	*Cinara*	*pilicornis*	*Picea*	Washington (US)	unknown	KF649502
3031	*Cinara*	*pilicornis*	*Picea*	Washington (US)	shoot	KF649507
1114	*Cinara*	*pinea*	*Pinus*	Languedoc-Roussillon (Fr)	shoot	KF649340
1262	*Cinara*	*pinea*	*Pinus*	Limousin (Fr)	unknown	KF649345
2163	*Cinara*	*pinea*	*Pinus*	Almaty (KZ)	shoot	KF649353

2265	*Cinara*	*pinea*	*Pinus*	Almaty (KZ)	shoot	KF649355
2669	*Cinara*	*pinea*	*Pinus*	PACA (Fr)	shoot	KF649361
2686	*Cinara*	*pinea*	*Pinus*	PACA (Fr)	shoot	KF649364
2723	*Cinara*	*pinea*	*Pinus*	Languedoc-Roussillon (Fr)	shoot	KF649373
2724	*Cinara*	*pinea*	*Pinus*	Languedoc-Roussillon (Fr)	shoot	KF649374
2728	*Cinara*	*pinea*	*Pinus*	Languedoc-Roussillon (Fr)	unknown	KF649378
2810	*Cinara*	*pinea*	*Pinus*	Almaty (KZ)	shoot	KF649391
2830	*Cinara*	*pinea*	*Pinus*	Almaty (KZ)	shoot	KF649396
2941	*Cinara*	*pinea*	*Pinus*	PACA (Fr)	branch	KF649463
3005	*Cinara*	*pinea*	*Pinus*	Oregon (US)	shoot	KF649494
3032	*Cinara*	*pinea*	*Pinus*	Washington (US)	shoot	KF649508
3039	*Cinara*	*pinea*	*Pinus*	Washington (US)	shoot	KF649512
3052	*Cinara*	*pinea*	*Pinus*	Oregon (US)	shoot	KF649521
1239a	*Cinara*	*pinea*	*Pinus*	Auvergne (Fr)	unknown	KF649342
1261b	*Cinara*	*pinea*	*Pinus*	Limousin (Fr)	unknown	KF649344
2682	*Cinara*	*pini*	*Pinus*	PACA (Fr)	branch	KF649363
2710	*Cinara*	*pini*	*Pinus*	PACA (Fr)	shoot	KF649371
2729	*Cinara*	*pini*	*Pinus*	PACA (Fr)	branch	KF649379
2932	*Cinara*	*pini*	*Pinus*	PACA (Fr)	branch	KF649459
2934	*Cinara*	*pini*	*Pinus*	PACA (Fr)	branch	KF649460
2935	*Cinara*	*pini*	*Pinus*	PACA (Fr)	branch	KF649461
1261a	*Cinara*	*pini*	*Pinus*	Limousin (Fr)	unknown	KF649343
2017	*Cinara*	*pinimaritimae*	*Pinus*	Sicily (It)	shoot	KF649352
3018	*Cinara*	*pinivora*	*Pinus*	Washington (US)	branch	KF649501
2863	*Cinara*	*ponderosae*	*Pinus*	Colorado (US)	shoot	KF649402

2867	*Cinara*	*ponderosae*	*Pinus*	Colorado (US)	shoot	KF649404
2871	*Cinara*	*ponderosae*	*Pinus*	Colorado (US)	branch	KF649407
2872	*Cinara*	*ponderosae*	*Pinus*	Colorado (US)	shoot	KF649408
2887	*Cinara*	*ponderosae*	*Pinus*	Colorado (US)	shoot	KF649420
2958	*Cinara*	*ponderosae*	*Pinus*	California (US)	shoot	KF649468
2997	*Cinara*	*ponderosae*	*Pinus*	California (US)	shoot	KF649489
3061	*Cinara*	*ponderosae*	*Pinus*	Oregon (US)	shoot	KF649526
3062	*Cinara*	*ponderosae*	*Pinus*	Oregon (US)	shoot	KF649527
3073	*Cinara*	*ponderosae*	*Pinus*	California (US)	branch	KF649536
3083	*Cinara*	*ponderosae*	*Pinus*	California (US)	shoot	KF649546
3091	*Cinara*	*ponderosae*	*Pinus*	California (US)	shoot	KF649552
2924b	*Cinara*	*ponderosae*	*Pinus*	NewMexico	shoot	KF649456
661	*Cinara*	*pruinosa*	*Picea*	Mid-Pyrenees (Fr)	shoot	KF649560
2726	*Cinara*	*pruinosa*	*Picea*	Languedoc-Roussillon (Fr)	branch	KF649376
2804	*Cinara*	*pruinosa*	*Picea*	Almaty (KZ)	branch	KF649388
2806	*Cinara*	*pruinosa*	*Picea*	Almaty (KZ)	branch	KF649390
2909	*Cinara*	*pruinosa*	*Picea*	Colorado (US)	branch	KF649440
2883	*Cinara*	*pruinosa*	*Picea*	Colorado (US)	branch	KF649417
2882	*Cinara*	*pseudotaxifoliae*	*Pseudotsuga*	Colorado (US)	branch	KF649416
2888	*Cinara*	*pseudotaxifoliae*	*Pseudotsuga*	Colorado (US)	branch	KF649421
2917	*Cinara*	*pseudotaxifoliae*	*Pseudotsuga*	New-Mexico (US)	shoot	KF649448
2919	*Cinara*	*pseudotaxifoliae*	*Pseudotsuga*	New-Mexico (US)	shoot	KF649450
2981	*Cinara*	*pseudotaxifoliae*	*Pseudotsuga*	California (US)	branch	KF649478
2999	*Cinara*	*pseudotaxifoliae*	*Pseudotsuga*	Oregon (US)	branch	KF649490
3001	*Cinara*	*pseudotaxifoliae*	*Pseudotsuga*	Oregon (US)	branch	KF649491

3029	*Cinara*	*pseudotaxifoliae*	*Pseudotsuga*	Washington (US)	branch	KF649506
3047	*Cinara*	*pseudotaxifoliae*	*Pseudotsuga*	Oregon (US)	N	KF649516
3056	*Cinara*	*pseudotaxifoliae*	*Pseudotsuga*	Oregon (US)	branch	KF649523
3076	*Cinara*	*pseudotaxifoliae*	*Pseudotsuga*	California (US)	branch	KF649539
2921	*Cinara*	*pseudotaxifoliae*	*Abies*	New-Mexico (US)	branch	KF649452
2881	*Cinara*	*pseudotsugae_sensu*_Palmer	*Pseudotsuga*	Colorado (US)	shoot	KF649415
2889	*Cinara*	*pseudotsugae_sensu*_Palmer	*Pseudotsuga*	Colorado (US)	branch	KF649422
2918	*Cinara*	*pseudotsugae_sensu*_Palmer	*Pseudotsuga*	New-Mexico (US)	branch	KF649449
2915bis	*Cinara*	*pseudotsugae_sensu*_Palmer	*Pseudotsuga*	New-Mexico (US)	N	KF649446
2890	*Cinara*	*pseudotsugae_sensu*_Palmer	*Pseudotsuga*	Colorado (US)	branch	KF649423
2895	*Cinara*	*puerca*	*Pinus*	Colorado (US)	trunk	KF649428
2968	*Cinara*	*puerca*	*Pinus*	California (US)	branch	KF649473
2877	*Cinara*	*schwartzi*	*Pinus*	Colorado (US)	branch	KF649412
2898	*Cinara*	*schwartzi*	*Pinus*	Colorado (US)	branch	KF649430
2971	*Cinara*	*schwartzi*	*Pinus*	California (US)	branch	KF649475
2901b	*Cinara*	*schwartzi*	*Pinus*	Colorado (US)	branch	KF649432
2924c	*Cinara*	*schwartzi*	*Pinus*	New-Mexico (US)	shoot	KF649457
3028	*Cinara*	*sitchensis*	*Picea*	Washington (US)	branch	KF649505
2862b	*Cinara*	*solitaria*	*Pinus*	Colorado (US)	shoot	KF649401
2870a	*Cinara*	*solitaria*	*Pinus*	Colorado (US)	shoot	KF649405
2987	*Cinara*	*splendens_sensu*_Palmer	*Pseudotsuga*	California (US)	shoot	KF649483
2996	*Cinara*	*splendens_sensu*_Palmer	*Pseudotsuga*	California (US)	shoot	KF649488
3004	*Cinara*	*splendens_sensu*_Palmer	*Pseudotsuga*	Oregon (US)	shoot	KF649493
3025	*Cinara*	*splendens_sensu*_Palmer	*Pseudotsuga*	Washington (US)	branch	KF649504
3077	*Cinara*	*splendens_sensu*_Palmer	*Pseudotsuga*	California (US)	branch	KF649540

3093	*Cinara*	*splendens_sensu_*Palmer	*Pseudotsuga*	California (US)	twig	KF649553
2955	*Cinara*	*terminalis*	*Pinus*	California (US)	shoot	KF649466
2964	*Cinara*	*terminalis*	*Pinus*	California (US)	shoot	KF649470
2966	*Cinara*	*terminalis*	*Pinus*	California (US)	shoot	KF649471
2858	*Cinara*	*terminalis*	*Pinus*	Colorado (US)	shoot	KF649400
2892	*Cinara*	*terminalis*	*Pinus*	Colorado (US)	shoot	KF649425
2922	*Cinara*	*terminalis*	*Pinus*	New-Mexico (US)	shoot	KF649453
2899bis	*Cinara*	*terminalis*	*Pinus*	Colorado (US)	shoot	KF649431
1969	*Cinara*	*tujafilina*	*Chamaecyparis*	Sicily (It)	shoot	KF649348
2462	*Cinara*	*tujafilina*	*Thuja*	Languedoc-Roussillon (Fr)	shoot	KF649358
2835	*Cinara*	*tujafilina*	*Thuja*	Languedoc-Roussillon (Fr)	shoot	KF649398
3009	*Cinara*	unknown	*Picea*	Oregon (US)	branch	KF649496
3011	*Cinara*	unknown	*Picea*	Washington (US)	shoot	KF649498
3017	*Cinara*	unknown	*Picea*	Washington (US)	branch	KF649500
3046	*Cinara*	unknown	*Picea*	Oregon (US)	branch	KF649515
3082	*Cinara*	unknown	*Picea*	California (US)	shoot	KF649545
2985	*Cinara*	unknown	*Picea*	California (US)	shoot	KF649482
2885b	*Cinara*	*vandykei*	*Picea*	Colorado (US)	branch	KF649419
2896	*Cinara*	*wahluca*	*Juniperus*	Colorado (US)	branch	KF649429
2893	*Cinara*	*wahtolca*	*Pinus*	Colorado (US)	branch	KF649426
2923	*Cinara*	*wahtolca*	*Pinus*	New-Mexico (US)	branch	KF649454
2975	*Cinara*	*watsoni*	*Pinus*	California (US)	shoot	KF649477
2982	*Cinara*	*watsoni*	*Pinus*	California (US)	shoot	KF649479
1101	*Lachnus*	*roboris*	*Quercus*	Languedoc-Roussillon(FR)	Not applicable	KF649339
1211	*Trama*	*troglodytes*	*Achillea*	Haute-Normandie (FR)	Not applicable	KF649341

2318	*Tuberolachnus*	*salignus*	*Salix*	Languedoc-Roussillon(FR)	Not applicable	KF649356

Figure S1. Distributions of the conifer-feeding aphids at the collection sites in the mountains of southwest China. This original map was created using ArcGIS 9.3 software.

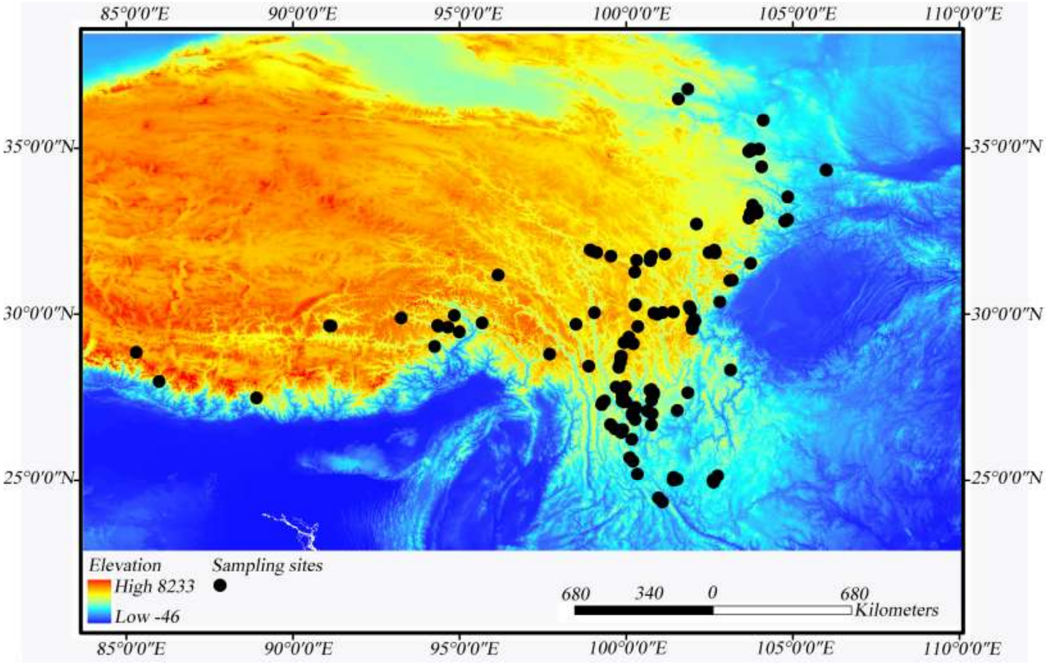

Supplement: Supplementary Information [file srep20123-s1.pdf]
